# A helminth–microbiota metabolic relay promotes disease tolerance

**DOI:** 10.1080/19490976.2026.2748132

**Published:** 2026-10-15

**Authors:** Ruohang Sun, Yi Liu, Yang Wang, Nuo Xu, Xiaoxiao Ma, Qingbo Lv, Xiufeng Zhao, Pascal Boireau, Zeming Wu, Mingyuan Liu, Xiaolei Liu, Xuemin Jin

**Affiliations:** a State Key Laboratory for Diagnosis and Treatment of Severe Zoonotic Infectious Diseases, Key Laboratory for Zoonosis Research of the Ministry of Education, Institute of Zoonosis, College of Veterinary Medicine, Jilin University, Changchun, China; b College of Food Science and Engineering, Jilin University, Changchun, China; c Würzburg Institute of Systems Immunology, Max Planck Research Group at the Julius-Maximilians University of Würzburg, Würzburg, Germany; d ANSES, Laboratory for Animal Health, Maisons-Alfort, France; e UMR BIPAR, Anses, Laboratoire de Santé Animale, INRAE, Ecole Nationale Vétérinaire d'Alfort, Maisons-Alfort, France; f School of Life Sciences, Jilin University, Changchun, China

**Keywords:** *Trichinella spiralis*, gut microbiota, ILA, AhR, Treg/Th17

## Abstract

Persistent infection by helminths requires limiting inflammatory damage despite incomplete pathogen clearance, yet how helminths mobilize resident microbiota to establish this state remains unclear. Here we identify a cross-kingdom metabolic relay through which *Trichinella spiralis* promotes systemic disease tolerance. Infection selectively expanded *Ligilactobacillus murinus* and redirected microbial tryptophan metabolism toward indole-3-lactic acid (ILA), a direct ligand of the aryl hydrocarbon receptor (AhR). Antibiotic perturbation abrogated ILA production, AhR activation and Treg–Th17 remodeling, whereas transfer of helminth-conditioned fecal preparations or supplementation with *L. murinus* or ILA restored these responses. Pharmacological inhibition or genetic deletion of AhR eliminated the protection conferred by *T. spiralis*, *L. murinus* and ILA against endotoxin-induced lung injury. In lethal SARS-CoV-2 infection, oral ILA improved survival and limited pulmonary immunopathology without reducing viral burden, thereby uncoupling host protection from pathogen control. In a human COVID-19 cohort, ILA relative abundance remained inversely associated with IFN-γ after adjustment for available clinical covariates. These findings reveal that helminths can reprogram commensal metabolism to govern systemic immune tolerance and identify microbial ILA production as a tractable postbiotic strategy for limiting inflammatory tissue damage.

## Graphical abstract

**Figure:**
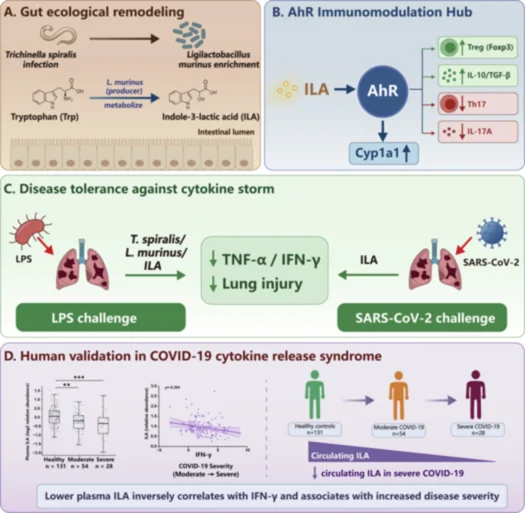

## Introduction

Persistent helminth infection poses a fundamental problem in host defense: how can immunity limit tissue damage without eliminating the invader? Helminths infect more than one billion people and often persist for years within immunocompetent hosts.^1^ They establish regulatory programs characterized by expansion of regulatory T cells (Tregs) and production of IL-10 and TGF-β.^2^ This co-evolved capacity underpins the “old friends” framework and has motivated efforts to harness helminth immunoregulation for inflammatory disease.^3-6^ Most mechanistic studies, however, have centered on parasite-derived molecules that act directly on host cells. Yet helminths occupy a densely colonized mucosal ecosystem, raising the possibility that they also impose systemic tolerance by reprogramming microbial metabolism. The intestinal microbiota contributes to helminth-mediated suppression of allergic inflammation,^7^ but the bacterial producers, metabolites, and host sensors that connect parasite exposure to immune protection remain unresolved.

Helminths inhabit a densely colonized mucosal ecosystem, and infection can reshape host-microbe interactions.^8^ ^,^ ^9^ Across experimental and human studies, these changes do not uniformly increase diversity; they reorganize community structure, enrich metabolically specialized taxa, and alter the pool of bioactive metabolites available to the host.^10-13^ Microbial tryptophan metabolism is a particularly compelling candidate pathway: intestinal bacteria convert dietary tryptophan into indole derivatives that function as interkingdom signals.^13^ ^,^ ^14^ Several of these metabolites engage the aryl hydrocarbon receptor (AhR), a ligand-responsive transcription factor that coordinates barrier integrity and immune homeostasis.^15-18^ AhR outputs are highly context dependent, varying with ligand chemistry, responding cell type, tissue site, and inflammatory state.^19^ ^,^ ^20^ In the adaptive immune compartment, this plasticity can tune the balance between Tregs and pro-inflammatory T helper 17 (Th17) cells. A decisive test of this model therefore requires a complete causal chain from a helminth-remodeled bacterial taxon, through a defined metabolite and host receptor, to a disease-relevant protective phenotype.

Here we identify such a circuit initiated by *Trichinella spiralis*. Infection enriches *Ligilactobacillus murinus*, whose culture supernatants show marked indole-3-lactic acid (ILA) accumulation; ILA directly engages AhR and biases the Treg/Th17 axis toward a regulatory state. Antibiotic perturbation, pharmacologic AhR blockade, and genetic AhR deficiency each abrogate this program, whereas fecal transfer and supplementation with either *L. murinus* or ILA recapitulate its key features. The resulting *T. spiralis*–*L. murinus*–ILA–AhR axis limits cytokine-storm-like lung injury. In lethal SARS-CoV-2 infection, oral ILA improves survival and suppresses pulmonary immunopathology without reducing viral burden, indicating enhanced disease tolerance. Reanalysis of a human COVID-19 cohort further supports an inverse association between circulating ILA and IFN-γ. Together, our data define a parasite–microbiota–metabolite–receptor pathway that promotes disease tolerance. This framework positions helminths as ecological engineers of host-associated microbial metabolism and ILA production as a tractable postbiotic principle for limiting inflammatory tissue injury.

## Materials and methods

### Ethics statement

C57BL/6J wild-type mice (6–8 weeks old) were purchased from Changchun Yisi Biotechnology Co., Ltd. C57BL/6J AhR-knockout mice (6 weeks old) and K18-hACE2 mice (5–6 weeks old) were obtained from GemPharmatech Co., Ltd. Mice were maintained under specific pathogen-free conditions on a 12-h light/dark cycle with free access to chow and water. All animal studies, including the euthanasia procedure, were approved by the Institutional Animal Care and Use Committee of Jilin University (Permit No. KT20230367) and were conducted in accordance with institutional guidelines and the ARRIVE recommendations. Mice were euthanized by carbon dioxide (CO₂) inhalation.

### Animals, helminth infection, and AhR inhibition

*Trichinella spiralis* (ISS534) muscle larvae were recovered from Wistar rats 35 d after oral infection with 5000 larvae. For the infection model, mice were orally inoculated with 250 muscle larvae and euthanized at 14 d post-infection (dpi). Where indicated, mice received the AhR antagonist CH223191 (10 mg/kg; MedChemExpress) by intraperitoneal injection during the infection or intervention period.

### Antibiotic-treated *T. spiralis* infection

For antibiotic perturbation experiments, mice were randomized into three groups (*n* = 6 per group): antibiotic only, *T. spiralis* only, and antibiotic plus *T. spiralis*. The antibiotic cocktail contained ampicillin (0.5 g/L), neomycin (0.5 g/L), metronidazole (0.5 g/L), and vancomycin (0.25 g/L) and was administered by daily oral gavage from day −14 to 14 dpi. Infected mice received 250 *T. spiralis* muscle larvae by oral gavage. All mice were euthanized at 14 dpi.

### Fecal microbiota transplantation

To test whether helminth-conditioned fecal preparations were sufficient to transfer the metabolic phenotype, mice were assigned to control, antibiotic-treated, antibiotic plus control-FMT, or antibiotic plus Ts-FMT groups (*n* = 6 per group). The process of FMT was performed as previously described.^21^ After 28 d of antibiotic treatment, recipient mice received fecal preparations from uninfected donors or from *T. spiralis*-infected donors at 14 dpi. Fresh fecal pellets were suspended immediately in sterile PBS (1 pellet/mL), homogenized, and centrifuged at 100 × *g* for 5 min at 4 °C. The supernatant was used as the transplant inoculum.

### *L. murinus* and ILA administration experiments

To confirm the role of dominant probiotics and key metabolite in helminth infection, *L. murinus* or ILA administration were performed. *L. murinus* (BNCC) was cultured in MRS medium and maintained in an anaerobic incubator using the GasPak 100 system (BD Bioscience) at 37 °C. After 21 d of ABX treatment, WT mice and AHR KO mice were gavaged with 200 ul of 1 × 10^9^ CFU/mL *L. murinus* every day for 7 d, and control mice were gavaged with 200 μl of blank MRS medium every day for 7 d. For the ILA treatment experiment, mice were gavaged with either 0.1 mL/20 g of corn oil (Solarbio) or 20 mg/kg of ILA (Med Chem Express) once a day for 7 d after 21 d of ABX pretreatment. For the AhR antagonist pretreatment experiments mice were pretreated with CH223191 at a dosage of 10 mg/kg 1 h before gavage of *L. murinus* or ILA.

### LPS-induced cytokine storm model

Acute sepsis models were induced by LPS from Escherichia coli 0111: B4 (L2630, Sigma-Aldrich), LPS dissolved in 200 μl sterile phosphate buffer, and intraperitoneally injected at a dose of 8 mg/kg into each mouse. Other groups of mice were intraperitoneally injected with equal volumes of sterile PBS. For the Ts pretreatment group, each mouse was orally administered 250 TsML and injected with LPS at 14 dpi. For the *L. murinus* and ILA pretreatment groups, each mouse was gavaged daily for 7 d with either 200 μL of *L. murinus* at 1 × 10^9^ CFU/mL or 20 mg/kg of ILA, followed by LPS injection after the final treatment. For the AhR antagonist pretreatment experiments mice were pretreated with CH223191 at a dosage of 10 mg/kg 1 h before gavage of Ts, *L. murinus* or ILA. Six hours after LPS injection, all mice were euthanized under anesthesia and samples were collected.

### ILA protection in hACE2 transgenic mice

All K18-hACE2 mice experiments were conducted in the biosafety protection level III Laboratory (ABSL-3) certified by China National Accreditation Service for conformity assessment (CNAS). The experimental work on SARS-CoV-2 virus has been approved by the National Health Commission. K18-hACE2 mice (5–6 weeks old) were randomly assigned to three groups: control group, SARS-CoV-2-infected group, and SARS-CoV-2 plus indole-3-lactic acid group (SARS-CoV-2+ILA). Mice in the SARS-CoV-2 group were intranasally infected with 5.0 × 10^4^ PFU of SARS-CoV-2 WIV04 strain (IVCAS 6.7512). Mice in the SARS-CoV-2+ILA group received ILA at 20 mg/kg by oral gavage once daily from 7 d before infection to 7 d after infection. Mice in the control and SARS-CoV-2 groups received an equal volume of vehicle. For longitudinal assessment, a subset of mice (*n* = 12) from each group was monitored for 14 d after infection, during which survival and body weight were recorded daily. Body weight change was expressed as the percentage of the initial body weight measured on the day of infection. A separate subset of mice (*n* = 3–5) was euthanized at 6 d post-infection (dpi) for virological, histopathological, and immunological analyses.

Viral titers in lung tissues were determined by plaque assay on Vero E6 cells as previously described.^22^ Briefly, the samples were serially ten-fold diluted using Dulbecco's modified Eagle's medium (DMEM) containing 2.5% fetal bovine serum (FBS) plus 2% penicillin/streptomycin, and 100 μL of each dilution was added to Vero E6 cells in 24-well-plates. After 1 h incubation of the plates, the inoculums were replaced by fresh methylcellulose overlay containing 2% FBS. The plates were incubated at 37 °C for 3 d, followed by fixation with 8% paraformaldehyde. Then, the plates were stained with 1% crystal violet, and visible plaques were counted to determine viral titers as plaque-forming units (PFU). For histopathology, mice were euthanized before harvest and fixation of tissues. Lungs were inflated with about 2 ml of 10% neutral buffered formalin using a 3-ml syringe and catheter inserted into the trachea and kept in fixative for 7 d. Tissues were embedded in paraffin, and sections were stained with hematoxylin and eosin. In parallel, lung homogenates were analyzed by ELISA to quantify inflammatory and regulatory cytokines, including TNF-α, IFN-γ, IL-10, TGF-β, and IL-17A, according to the manufacturers' instructions.

### Gene expression analysis

As described above, quantitative real-time PCR (qPCR) was used to measure mRNA expression levels of genes in the intestine and organoids.^23^ Total RNA from intestine and organoids was isolated with TRIzol followed by sequential chloroform, isopropanol, and 75% ethanol treatments under RNase-free conditions. Prime Script RT Master Mix (Takara) served for cDNA synthesis. Quantitative RT-PCR was performed using SYBR Green qPCR Master Mix Kit (Genstar) on a Step One Plus instrument (Applied Biosystems). Gene expression was normalized to β-actin and calculated using the 2^−ΔΔCT^ method. Oligonucleotide sequences are provided in Table S1.

### Lung histology

Lung tissues were immediately fixed in 4% paraformaldehyde (≥24 h), paraffin-embedded, and sectioned at 5 μm. Sections underwent hematoxylin and eosin (H&E) staining prior to light microscopic examination. Histopathological evaluation and lung injury scoring were performed independently by an investigator blinded to the experimental group allocation. Lung injury severity was scored per established criteria,^24^ assessing four parameters: pulmonary hemorrhage, alveolar congestion, and neutrophil infiltration in vessel walls or airspaces. Lung injury severity was scored 0–4: 0 (minimal), 1 (mild), 2 (moderate), 3 (severe), 4 (maximal). A composite lung injury score was generated by aggregating these four metrics.

### Cytokines assays in serum

Blood and organoid culture supernatants were collected from mice retro-orbital plexuses underwent centrifugation (3000 rpm, 15 min, 4 °C), with serum analyzed for cytokines using ELISA kits (Elabscience).

### Quantification of fecal bacterial load

Total bacterial DNA was extracted from fecal samples using a Fecal Genomic DNA Extraction Kit (TIANGEN) according to the manufacturer's instructions. Bacterial 16S rRNA gene copies were quantified by qPCR using the universal bacterial primer pair 16S-341_F (5′-CCTACGGGNGGCWGCAG-3′) and 16S-805_R (5′-GACTACHVGGGTATCTAATCC-3′). qPCR was performed using SYBR Green qPCR Master Mix (Genstar) on a Step One Plus instrument (Applied Biosystems).

### Flow cytometry

Spleen and mesenteric lymph nodes (MLNs) were aseptically harvested. Single-cell suspensions were generated by gentle mechanical dissociation through cell strainer (70 μm) in ice-cold PBS, followed by cell counting and density adjustment to 1 × 10^6^/mL. Erythrocyte lysis was performed with red blood cell lysis buffer (Solarbio) for 10 min. Cells underwent 4 h stimulation at 37 °C in 5% (fetal bovine serum) FBS-RPMI-1640 with PMA/ionomycin cocktail plus protein transport inhibitors (BFA, Biolegend) for Th17 intracellular cytokine staining. Cells were then washed twice in FACS buffer (PBS with 2% FBS), blocked for 15 min in FACS buffer containing anti-mouse CD16/32 antibody, and incubated with Fixable Viability Dye eFluor™ 780 and surface antibodies (FITC-anti-CD4, Biolegend; and APC-anti-CD25 antibody, BD Biosciences) at 4 °C for 30 min in the dark. Cells were then washed twice in FACS buffer and incubated in Fix/Perm solution (eBioscience) for 30 min at 4 °C in the dark. Cells were then washed twice in permeabilization buffer and incubated with intracellular antibodies (PE-anti-IL-17A, Biolegend; and PE-anti-Foxp3, eBioscience) for 30 min at 4 °C in the dark. Flow cytometric data acquisition used BD FACSCalibur flow cytometer (BD Biosciences), with subsequent analysis in FlowJo software (Tree Star Inc, Ashland, OR).

### Western blot analysis

Proteins were extracted from intestine tissue using PBS buffer containing protease inhibitors. After quantification using BCA Protein Assay Kit, equivalent quantities were resolved on 10%–12% gradient SDS-PAGE and electrotransferred to nitrocellulose (NC) membranes. After being blocked in 5% skim milk for 2 h at room temperature, the NC membranes were incubated overnight at 4 °C with primary antibodies (anti-AhR/Cyp1a1 (1:1000) and anti-GAPDH (1:10,000)). Wash the NC membrane with TBST three times, and then incubate it with the secondary antibody at room temperature for 1 h. Finally, the membranes were identified using an enhanced chemiluminescence (ECL) kit.

### Intestinal organoid isolation and treatments

The mice intestinal organoid isolation and treatments was performed as described below.^25^ Briefly, Intestinal segments of 5–10 cm were aseptically harvested from 4–6-week-old C57BL/6 wild-type mice. Tissues were longitudinally incised, repeatedly flushed with cold PBS, and villi mechanically scraped using sterile glass slides. Cut the intestine into pieces of 1–2 cm and wash repeatedly with cold PBS. Tissue fragments underwent 30 min incubation in 5 mM EDTA-PBS solution at 4 °C on a rotary shaker. Crypts were subsequently dislodged from the basement membrane by vigorous shaking. Following EDTA dissociation, crypts were enriched in supernatants, filtered through 70 μm cell strainers, and pelleted by centrifugation for 5 min. Intestinal organoids were cultured at 37 °C in a 5% CO_2_ atmosphere.

For *L. murinus* and ILA treatment experiments, organoids were cultured in *L. murinus* (1 × 10^6^ CFU/mL), ILA (2 mM), CH223191 (10 μM), LPS (200 μg/mL) for 48 h. The organoids were randomized into five groups before being subjected to LPS experiments: (1) Con. (2) LPS. (3) *L. murinus*+LPS. (4) *L. murinus*+LPS+CH223191 (5). ILA+LPS (6) ILA+LPS+CH223191. For Immunofluorescence experiment, organoids were fixed in 4% paraformaldehyde for 1 h at 4 °C, dehydrated through 15%–30% sucrose for 48 h. Organoids sections were stained with primary antibodies (anti-AhR/Cyp1a1 (1:1000)).

### Molecular docking and molecular dynamics (MD) simulation

The structure of the human AhR (Homo sapiens) used for molecular docking and subsequent MD simulation was retrieved from the Protein Data Bank. Protein and small molecule structure files were imported into GROMACS 2022.3.^26^ Topology and simulation boxes were generated using pdb2gmx and gmx editconf, respectively. Energy minimization was performed with the steepest descent algorithm. MD simulations were conducted at 300 K and 1 bar using the Amber99sb-ildn force field. Equilibration consisted of 100,000 steps under the NVT ensemble, followed by 100,000 steps under the NPT ensemble, both with a coupling constant of 0.1 ps and a duration of 100 ps. Production MD simulations were run for 100 ns using gmx grompp and gmx mdrun, during which conformational changes were recorded. Trajectories were analyzed using built-in GROMACS tools to calculate root mean square deviation (RMSD), root mean square fluctuation (RMSF) per residue, and the free energy landscape.

### Surface plasmon resonance (SPR) analysis

The binding affinity between ILA and recombinant human AhR was assessed using a Biacore X100 system with a CM5 sensor chip (Cytiva). AhR protein, diluted in acetate buffer (pH 4.0), was immobilized on the chip following surface activation with EDC and NHS. Serial concentrations of ILA (3.125, 6.25, 12.5, 25, and 50 μg/mL) were prepared in running buffer (PBS containing Tween-20) and injected for kinetic measurements. Sensorgrams were processed and analyzed using the Biacore Evaluation software.

### Targeted metabolomics of tryptophan metabolites

Targeted metabolomic analysis of tryptophan metabolites was performed by Novogene. Co., Ltd. using targeted metabolomics. All standards, including stable isotope-labeled analogs, were sourced from ZZStandards Co., LTD. Methanol (Optima LC-MS), acetonitrile (Optima LC-MS), formic acid (Optima LC-MS) and Ammonium acetate (Optima LC-MS) were purchased from Thermo-Fisher Scientific. Imino-bis (methylphosphonic acid) was purchased from Sigma-Aldrich. Ultrapure water was purchased from Millipore. In brief, the stock solution of individual CCM related compounds was mixed and prepared in CCM related compounds-free matrix to obtain a series of calibrators. The samples were resuspended with liquid nitrogen, and then added to water by well vortexing as the diluted sample. Then 100 μL of them were taken respectively and homogenized with 300 μL of methanol (80%) which contained mixed internal standards by well vortexing. Next, stewing for 10 min. After that, centrifuged at 15,000 *g* for 15 min. Finally, the supernatant was injected into the LC-MS/MS system for analysis. Absolute metabolite concentrations were reported as ng/g for fecal samples and as ng/mL for serum and bacterial-culture supernatants.

The metabolite abundance data were normalized (log-transformation and Pareto scaling) before PCoA to reduce bias from highly abundant metabolites. Heatmaps were generated using the pheatmap package in R (v4.2.1) to visualize differential metabolite patterns across samples. Hierarchical clustering was applied using the Bray–Curtis distance metric and complete linkage method.

### Shotgun metagenomic sequencing and taxonomic analysis

As for metagenomic sequencing data (PRJNA1321505), raw sequencing reads were processed to obtain valid reads as previously described.^27^ Total genomic DNA was extracted from fecal samples using QIAamp DNA Stool Mini Kit (Qiagen). DNA integrity, sizes and concentrations were determined by agarose gel electrophoresis and NanoDrop spectrophotometry (NanoDrop). Sequencing libraries were constructed as previously described. After library quality control, high-throughput sequencing was performed using the NovaSeq6000 platform (Illumina).

Fastp is used for preprocessing raw data from the Illumina sequencing platform to obtain clean data for subsequent analysis.^28^ MEGAHIT software is used for assembly analysis of clean data.^29^

With the default parameters, MetaGeneMark is used to perform ORF prediction for scaftigs (≥500 bp) of each sample,^30^ and the information with a length less than 100 nt in the prediction results is filtered out.^31^ For the ORF prediction results, CD-HIT software is used to eliminate redundancy^32^ and obtain the nonredundant initial gene catalogue. Clean data of each sample is aligned to the initial gene catalogue by using Bowtie2 to calculate the number of reads of the genes on each sample alignment.^33^ Based on the abundance of each gene in the gene catalogue in each sample, basic information statistics, core-pan gene analysis, correlation analysis between samples, and Venn diagram analysis of gene number are performed.

DIAMOND software is used for alignment of unigenes sequences with Micro NR database, which includes sequences from bacteria, fungi, archaea, and viruses extracted from NCBI's NR database. The alignment is performed using the BLASTP algorithm with a parameter setting of 1e^−5^.​​​​^34^ Since each sequence may have multiple alignment results, LCA algorithm applied to systematic taxonomy of MEGAN software was adopted to determine the species annotation information of the sequence.^35^ Out of the results of LCA annotation and gene abundance table, the abundance of each sample at each taxonomy and the corresponding gene abundance tables are acquired. The abundance of a species in a sample is equal to the sum of the abundance of those genes annotated as that species. The number of genes of a species in a sample is equal to the number of genes whose abundance is non-zero among the genes annotated as that species. LEfSe software is used for LEfSe analysis.

### Human COVID-19 cohort reanalysis

We reanalyzed clinical and plasma data from Su et al.^36^ verified against Mendeley Data version 5 (doi:10.17632/tzydswhhb5.5). Exact sample identifiers paired ILA (indolelactate) with IFN-γ; patient and verified draw identifiers linked clinical records. The primary population had concurrent WHO Ordinal Scale (WOS) 3–7: moderate, 3–4; severe, 5–7. Clinical models used log2 ILA relative abundance; Olink IFN-γ NPX, already log2-scaled, was unchanged. Missing measurements were not imputed.

Gaussian identity-link generalized estimating equations (GEE) used patient clusters, exchangeable working correlation and robust sandwich covariance. M0 was unadjusted; M1 included age and sex; M2 (core clinical adjustment) added draw-specific BMI, T1/T2 visit and a comorbidity count comprising hypertension, type 2 diabetes, coronary artery disease, and chronic pulmonary disease (asthma or COPD). All four components required recorded data; missing diagnoses were not treated as absent. M3 additionally conditioned on concurrent severity. Models shared a complete-case population; maximum-available analyses were reported separately. Strict T1-only analyses used HC3-robust linear regression with t-based inference, omitted the constant visit term and never substituted T2. An all-COVID-19 sensitivity analysis included WOS 1–7. Patient-cluster bootstrap intervals (2000 replicates) assessed M2 and descriptive Spearman ρ; no independent-row Spearman *p-*value was used. Residual and leave-one-patient-out analyses assessed model stability; primary models retained all valid observations.

Figure 6L compared independent healthy donors and true T1 patient samples using three Welch tests on log2 ILA with Holm correction. Figure 6M plots source ILA relative abundance against IFN-γ NPX, with an unadjusted ordinary least-squares mean line and pointwise 95% confidence band from 2000 patient-cluster bootstrap refits (seed 20260921). The clinical analysis plan was fixed during revision before re-estimation, with the original unadjusted result known; the display fit was added subsequently.

### Statistical analysis

GraphPad Prism 8.0 was used for statistical analyses and data visualization. Two-group comparisons were performed with unpaired Student's *t*-tests, and comparisons among three or more groups were performed with one-way analysis of variance as appropriate. For metabolomic and metagenomic analyses, multiple-testing correction was performed with the Benjamini–Hochberg method. A two-sided *p*-value < .05 was considered statistically significant.

## Results

### Ts infection remodeled tryptophan metabolism to modulate the Treg/Th17 balance via AhR

Tryptophan, an essential amino acid, is metabolized through three major pathways: the Kynurenine Pathway, the Serotonin Pathway, and the Indole Derivative Pathway (Figure 1A).^15^ To investigate whether Ts infection affects host tryptophan metabolism, we performed targeted metabolomic profiling of tryptophan and its derivatives in infected mice. Principal Coordinates Analysis (PCoA) revealed a distinct separation in the metabolic profiles of fecal and serum samples between infected and control mice (Figure 1B; Figure S1A), indicating a significant metabolic shift driven by the infection. The heatmap shows the relative changes in metabolite concentrations between the control group and the infection group (Figure 1C; Figure S1B; Table 1). We observed that Ts infection led to a significant accumulation of tryptophan in the intestine (Figure S1C), alongside a concurrent decrease in serum tryptophan levels (Figure S1D; Table 2). This inverse relationship suggested enhanced local tryptophan metabolism within the gut. Indeed, analysis of the three major metabolic pathways revealed that metabolites of the indole derivative pathway were significantly elevated in the feces of infected mice. Fecal levels of indole, ILA, indole-3-aldehyde (IAld), and indole-3-acetic acid (IAA) were significantly elevated. Concomitantly, serum levels of ILA were also significantly increased compared to controls (Figure S2A–M). At 14 dpi, fecal ILA increased from 430.66 to 2060.17 ng/g (~4.78-fold, *p* = 0.0043), while serum ILA increased from 154.40 to 284.06 ng/mL (~1.84-fold, *p* = 0.026) (Figure S2; Tables 1 and 2). These data indicate that Ts infection preferentially redirects gut tryptophan metabolism toward the indole-derivative pathway, with ILA emerging as a prominent infection-associated metabolite.

**Table 1.** Fecal targeted tryptophan metabolites in control and Ts-infected mice.

Name	HMDB	Control (ng/g)	Ts (ng/g)	*p*-value	FC
2-Amino-3-methoxybenzoic acid	HMDB0060374	16.0800	5.4833	0.2133	0.3410
2-Oxoadipate	HMDB0000225	7168.0200	9160.4667	0.6623	1.2780
2-Oxoindole-3-acetate	HMDB0035514	50722.7000	46282.4500	0.5368	0.9125
3-Hydroxyanthranilic acid	HMDB0001476	162.3000	427.9167	0.3602	2.6371
3-Hydroxy-DL-kynurenine	HMDB0011631	316.7400	476.7667	0.4286	1.5053
3-Indoleacetic acid	HMDB0000197	2166.5000	3742.4500	0.0043	1.7274
3-Indoleacrylic acid	HMDB0000734	18.5400	15.8833	1.0000	0.8564
3-Indoleglyoxylic acid	HMDB0242143	498.2400	1555.6000	0.0303	3.1222
3-Indolepropionic acid	HMDB0002302	1242.6600	1242.2500	1.0000	0.9997
3-Methylindole	HMDB0000466	14460.9800	14941.2667	0.9307	1.0332
4,6-Dihydroxyquinoline	HMDB0004077	80.7600	43.4833	0.5368	0.5388
5-Hydroxyindolacetic acid	HMDB0000763	465.6000	248.6833	0.0519	0.5341
5-Hydroxytryptophol	HMDB0001855	173.7800	113.6500	0.0519	0.6540
5-Methoxyindoleacetate	HMDB0004096	16.8800	8.6000	0.5830	0.5095
Alanine	HMDB0000161	263526.3000	254010.2500	0.9307	0.9639
Cinnabarinic acid	HMDB0004078	201.9800	153.9333	0.2468	0.7621
Formylanthranilate	HMDB0004089	741.8600	1194.8333	0.3290	1.6106
Formyl-N-acetyl-5-methoxykynurenamine	HMDB0004259	79.0400	53.8000	0.7922	0.6803
Indole	HMDB0000738	96.7600	271.1333	0.0043	2.8019
Indole-3-acetamide	HMDB0029739	8.5200	6.1833	0.6623	0.7258
Indole-3-carboxaldehyde	HMDB0029737	94.8400	166.5500	0.0043	1.7563
Indole-3-carboxylic acid	HMDB0003320	341.2600	446.7833	0.5368	1.3092
Indole-3-ethanol	HMDB0003447	1711.4400	1537.6500	0.9307	0.8984
Indole-3-lactic acid	HMDB0000671	430.6600	2060.1667	0.0043	4.7838
Indole-3-pyruvic acid	HMDB0060484	84.5800	96.6667	0.7739	1.1429
Indoxyl sulfate	HMDB0000682	1164.6200	14093.8333	0.7922	12.1017
Kynurenic acid	HMDB0000715	2577.5400	4345.3167	0.2468	1.6858
L-Kynurenine	HMDB0000684	249.2000	236.4667	1.0000	0.9489
L-Tryptophan	HMDB0000929	61350.3600	115036.5000	0.0087	1.8751
Melatonin	HMDB0001389	1.1800	0.9333	0.3580	0.7875
N-(3-Indolylacetyl)-L-alanine	HMDB0304378	44.1400	27.6000	0.2923	0.6259
N-Acetyl-5-hydroxytryptamine	HMDB0001238	120.0200	59.9500	0.0823	0.4995
Nicotinic acid	HMDB0001488	45004.7400	25831.2833	0.2468	0.5740
Picolinic acid	HMDB0002243	68551.6200	59280.8500	0.9307	0.8648
Quinolinic acid	HMDB0000232	2778.7400	1134.9167	0.2468	0.4084
Tryptamine	HMDB0000303	1183.1000	1853.3667	1.0000	1.5665
Xanthurenic acid	HMDB0000881	1844.4800	3137.7333	0.4286	1.7012

**Table 2.** Serum targeted tryptophan metabolites in control and Ts-infected mice.

Name	HMDB	Control (ng/mL)	Ts (ng/mL)	*p*-value	FC
Indoxylsulfate	HMDB0000682	5844.5779	9484.0288	0.3939	1.6227
5-Hydroxyindoleacetic acid	HMDB0000763	119.1303	149.1278	0.1797	1.2518
N-acetylserotonin	HMDB0001238	5.4704	4.4062	0.5887	0.8055
3-Indoleacrylic acid	HMDB0000734	4.5310	18.7826	0.0043	4.1453
Xanthurenic acid	HMDB0000881	22.6163	8.8625	0.0022	0.3919
Indole-3-lactic acid	HMDB0000671	154.3958	284.0603	0.0260	1.8398
Indolylpropionic acid	HMDB0002302	402.6000	841.1525	0.0087	2.0893
L-tryptophan	HMDB0000929	25176.1464	11151.8267	0.0022	0.4430
β-Indole-3-acetic acid	HMDB0000197	86.9914	149.5702	0.2403	1.7194
Hydroxytryptophan	HMDB0000472	60.1390	46.8825	0.1320	0.7796
Serotonin	HMDB0000259	2074.4299	1810.3482	0.8182	0.8727
2-Ketoadipic acid	HMDB0000225	430.7010	184.7024	0.0087	0.4288
3-Hydroxykynurenine	HMDB0000732	13.5827	9.7941	0.0931	0.7211
L-kynurenine	HMDB0000684	151.6479	261.9623	0.0411	1.7274
Nicotinic acid	HMDB0001488	31.9785	26.5733	0.1320	0.8310
2-Aminobenzoic acid	HMDB0001123	6.4422	10.4118	0.0649	1.6162
Indole-3-acetamide	HMDB0029739	8.1693	1.3977	0.0022	0.1711
Indole-3-carbaldehyde	HMDB0029737	20.8223	5.2479	0.0022	0.2520
Picolinic acid	HMDB0002243	10.5261	6.9685	0.0649	0.6620
N-Formylanthranilic acid	HMDB0004089	3.1584	1.8379	0.2403	0.5819
N-Formylkynurenine	HMDB0001200	6.8620	1.6273	0.0048	0.2371

**Figure 1.**
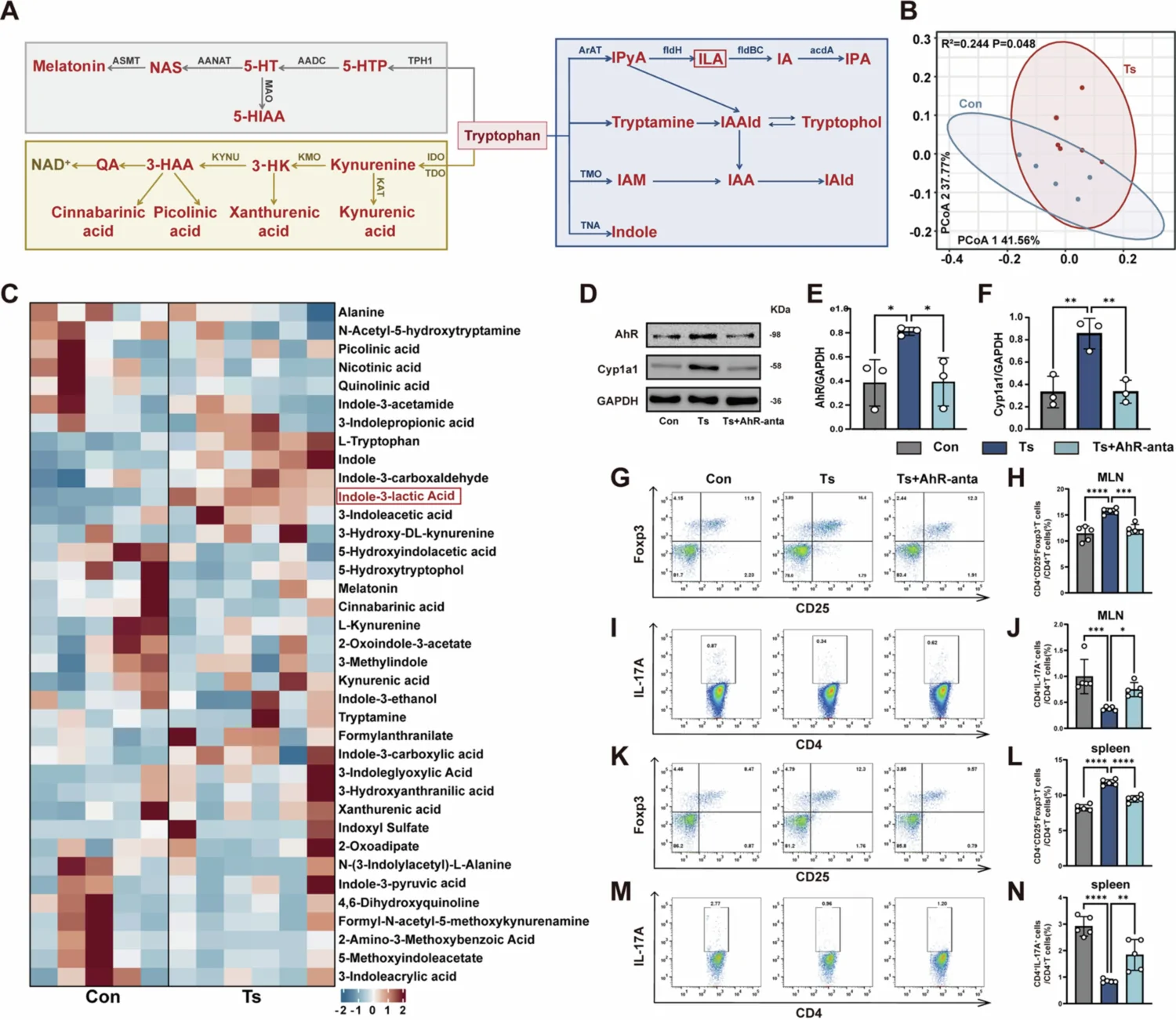
Ts infection remodels tryptophan metabolism to drive AhR-dependent modulation of the Treg/Th17 balance. (A) Schematic of the three major tryptophan metabolism pathways. (B) Principal coordinates analysis (PCoA) of fecal metabolite profiles from control and Ts-infected mice. (C) Heatmap of differentially abundant fecal metabolites. (D–F) Representative Western blot (D) and quantification (E, F) of AhR and Cyp1a1 protein level in duodenum tissues (*n* = 3). (G–N) Flow cytometric analysis of Treg (CD4^+^CD25^+^Foxp3^+^) and Th17 (CD4^+^IL-17A^+^) cells in mesenteric lymph nodes (G to J) and spleen (K to N) from control, Ts-infected, and Ts-infected plus CH223191-treated mice (*n* = 5 per group). Gating strategies are shown in Figure S4. Data are representative of three independent experiments. Data are presented as mean ± SD. Statistical analysis was performed using one-way ANOVA with Tukey's multiple (E, F, H, J, L and N). *p < 0.05, **p < 0.01, ***p < 0.001, ****p < 0.0001. Abbreviations: (IDO, indoleamine 2,3-dioxygenase; TDO, tryptophan 2,3-dioxygenase; KMO, kynurenine 3-monooxygenase; 3-HK, 3-hydroxykynurenine; KYNU, kynureninase; 3-HAA, 3-hydroxyanthranilic acid; QA, quinolinic acid; NAD⁺, Nicotinamide adenine dinucleotide (oxidized form); TPH1, tryptophan hydroxylase 1; AADC, aromatic L-amino acid decarboxylase; AANAT, arylalkylamine N-acetyltransferase; ASMT, Acetylserotonin O-methyltransferase; 5-HTP, 5-hydroxytryptophan; 5-HT, 5-hydroxytryptamine; MAO, monoamine oxidase; 5-HIAA, 5-hydroxyindoleacetic acid; NAS, N-acetylserotonin; ArAT, aromatic amino acid transaminase; fldH, phenyllactate dehydrogenase; fldBC, phenyllactyl-CoA dehydratase subunits B and C; acdA, aryl-CoA dehydrogenase A; TMO, tryptophan monooxygenase; TNA, tryptophanase; IPYA, indole-3-pyruvic acid; IPA, indole-3-propionic acid; IA, indole-3-acrylic acid; IAAld, indole-3-acetaldehyde; IAM, indole-3-acetamide; IAA, indole-3-acetic acid; IAld, indole-3-aldehyde.

Tryptophan metabolites can act as endogenous ligands to activate the AhR. As a key physiological regulator of immune homeostasis, AhR activation modulates the balance between Treg and Th17 cells.^37^ Disruption of the Treg/Th17 cells balance is strongly associated with autoimmune diseases and systemic inflammation. We explored whether Ts can activate the AhR signaling pathway. AhR protein and mRNA expression levels were significantly elevated in intestine of mice infected with Ts. Cytochrome P450 Family 1 Subfamily A Member 1 (Cyp1a1) serves as both a key downstream effector of AhR and a classical biomarker for AhR activation. Further analysis of Cyp1a1 expression revealed that Ts infection significantly upregulated both protein and mRNA levels of Cyp1a1. (Figure 1D–F; Figure S3A and B). We next investigated if Ts infection modulates host immunity via the AhR signaling pathway. Compared to controls, Ts infection significantly increased the mRNA expression of the Treg-associated transcription factor Foxp3 and the immunosuppressive cytokine TGF-β, while downregulating the Th17-associated effector cytokine IL-17A (Figure S3C–E). Pharmacological AhR blockade reversed these transcriptional and cellular changes, supporting an AhR-dependent contribution to the Ts-induced shift in the Treg/Th17 balance. Furthermore, flow cytometric analysis of lymphocyte subsets in the mesenteric lymph nodes (MLNs) and spleen revealed that the proportion of CD4⁺CD25⁺Foxp3⁺ Treg cells was significantly increased, while the proportion of CD4⁺IL-17A⁺ Th17 cells was markedly decreased in the infected group (Figure 1G–N; Figure S4). AhR antagonist treatment reversed these changes, confirming the central role of the AhR pathway in this immunomodulation. Because ILA abundance, AhR-Cyp1a1 activation, and Treg/Th17 phenotypes were assessed at the same 14-dpi endpoint, these observations define a coordinated infection-associated state.

### Ts-induced alteration in tryptophan metabolism was microbiota dependent

The gut microbiota has been confirmed as a critical modulator of tryptophan metabolism.^38^ To verify the mediating role of the gut microbiota, we established an antibiotic-treated (ABX) mouse model followed by Ts infection (Figure 2A). Quantification of fecal 16S rRNA gene copies confirmed effective microbiota depletion in antibiotic-treated mice (Figure S5A). Metagenomic profiling showed that ABX treatment reduced *α*-diversity and markedly altered β-diversity(Figure S5B–D). In contrast to microbiota-intact mice, Ts infection failed to increase fecal tryptophan-derived indoles in ABX-treated mice; fecal tryptophan and ILA levels remained low and were not significantly different between ABX and ABX+Ts groups (Figure 2B and C; Figure S5E and F; Table 3). Meanwhile, Ts infection failed to activate the AhR signaling pathway in ABX-treated mice, as no infection-induced upregulation of AhR or Cyp1a1 was observed in the intestinal tissue (Figure 2D–F; Figure S5G and H). Crucially, antibiotic treatment abolished the Ts-induced shift in the Treg/Th17 balance (Figure 2G–N; Figure S5I–K), supporting a critical microbiota-dependent component in this response. To determine whether antibiotic treatment altered parasite burden during the intestinal phase, we quantified intestinal adult *T. spiralis* in parallel. Adult worm burdens were comparable between Ts and ABX+Ts mice, with no significant difference detected (Figure S5L). Thus, the antibiotic-associated changes in ILA abundance, AhR/Cyp1a1 activation, and Treg/Th17 responses occurred without a detectable difference in adult parasite burden. To test whether Ts-associated regulatory activity was transferable with donor fecal preparations, we performed a fecal transfer experiment (Figure 2O). Fecal preparations from uninfected control donors or donors infected with Ts for 14 d were transferred into ABX recipient mice. We confirmed that there was no worm burden in the intestine of Ts-infected mice at 14 d post infection, as we have documented in previous study.^39^ ^,^ ^40^ Microscopic examination confirmed that no live adult worms or larvae were detectable in the fecal inocula used for FMT (data not shown). Because the inoculum was not sterile-filtered or otherwise fractionated, this experiment demonstrates that Ts-associated regulatory activity is transferable with the donor fecal preparation but does not formally exclude a contribution from soluble parasite-derived components. The results showed that no significant differences in the expression levels of AhR and Cyp1a1 were observed in the FMT-con group compared with the control group, while the expression levels of AhR and Cyp1a1 were significantly increased in the FMT-Ts group compared with both the control and FMT-con groups (Figure 2P–R; Figure S5M and N). Moreover, mRNA expression of Foxp3 and TGF-β was markedly upregulated, while IL-17A expression was significantly downregulated compared to the control group (Figure S5O–Q). Flow cytometric analysis showed that, compared with the FMT-Con group, the FMT-Ts group had significantly increased proportions of CD4⁺CD25⁺Foxp3⁺ Treg cells in the MLNs and spleen, while the proportions of CD4⁺IL-17A⁺ Th17 cells were significantly decreased (Figure 2S–Z). Collectively, these findings show that Ts-associated regulatory activity is transferable with donor fecal preparations and, together with the antibiotic and defined supplementation experiments, support a critical microbiota–metabolite contribution to the Treg/Th17 immune program. Because Ts infection was associated with increased indole metabolites (Figure 1; Figure S2), we next sought to identify the bacterial taxa linked to this metabolic shift.

**Table 3.** Fecal targeted tryptophan metabolites in ABX and ABX+Ts mice.

Name	HMDB	ABX (ng/g)	ABX + Ts(ng/g)	*p*-value	FC
Alanine	HMDB0000161	69117.5459	64911.3635	0.6623	1.2511
L-Tryptophan	HMDB0000929	6527.3996	6623.7212	1.0000	1.0019
L-Kynurenine	HMDB0000684	280.3456	419.7025	0.0173	1.4971
3-Indolepropionic acid	HMDB0002302	43.3563	50.5600	0.5368	1.1662
3-Hydroxy-DL-kynurenine	HMDB0011631	392.7803	444.7782	0.1775	1.1324
Nicotinic acid	HMDB0001488	6611.5928	4304.0672	0.0087	0.6510
Kynurenic acid	HMDB0000715	331.0456	925.2977	0.0043	2.7951
5-Hydroxyindolacetic acid	HMDB0000763	256.1505	323.5625	0.7922	1.2632
3-Indoleacetic acid	HMDB0000197	164.7936	91.5851	0.1775	0.5558
Xanthurenic acid	HMDB0000881	1614.7126	1254.3929	0.1255	0.7769
N-Formylkynurenine	HMDB0001200	504.8974	780.1546	0.4286	1.5452
Picolinic acid	HMDB0002243	848.2722	937.6252	0.3290	1.1053
N-Acetyl-5-hydroxytryptamine	HMDB0001238	1.3032	1.6304	0.6623	1.2511
Indole-3-acetamide	HMDB0029739	0.5732	0.5743	1.0000	1.0019
Indole	HMDB0000738	144.6844	109.2351	0.2468	0.7550
Indole-3-ethanol	HMDB0003447	187.6695	291.1229	0.3290	1.5513
Nicotinamide	HMDB0001406	85.7041	99.3099	0.3290	1.1588
5-Methoxytryptamine	HMDB0004095	2.1415	1.4734	0.1699	0.6880
3-Methylindole	HMDB0000466	732.0122	466.9759	0.6623	0.6379
6-Hydroxymelatonin	HMDB0004081	1.3079	1.7711	0.4102	1.3542
Melatonin	HMDB0001389	1.2139	0.7837	0.1699	0.6456
Tryptamine	HMDB0000303	5.6572	6.9891	0.2468	1.2354
Serotonin	HMDB0000259	1305.1474	1736.3428	0.3290	1.3304
5-Hydroxytryptophol	HMDB0001855	165.1548	263.3214	0.0303	1.5944
N-Methyltryptamine	HMDB0004370	0.1869	0.1960	0.9271	1.0488
Indole-3-carboxylic acid	HMDB0003320	104.0676	78.6972	0.1255	0.7562
Indole-3-lactic acid	HMDB0000671	789.1117	493.1209	0.1775	0.6249
3-Hydroxyanthranilic acid	HMDB0001476	556.4710	500.8588	0.9271	0.9001
Quinolinic acid	HMDB0000232	520.1405	340.2686	0.1255	0.6542
2-Oxoadipate	HMDB0000225	11766.3206	14093.0560	0.1775	1.1977
Formylanthranilate	HMDB0004089	83.7359	102.9958	0.3290	1.2300
3-Indoleglyoxylic acid	HMDB0242143	482.9229	399.7825	0.2468	0.8278
Formyl-N-acetyl-5-methoxykynurenamine	HMDB0004259	22.5244	7.2069	0.5368	0.3200
Indole-3-carboxaldehyde	HMDB0029737	63.1553	42.3842	0.0519	0.6711
Indole-3-pyruvic acid	HMDB0060484	98.8899	87.7484	0.4286	0.8873
2-Oxoindole-3-acetate	HMDB0035514	7294.9272	3949.2349	0.5368	0.5414
Indoxyl sulfate	HMDB0000682	2713.3128	217.4851	0.3290	0.0802
4,6-Dihydroxyquinoline	HMDB0004077	112.7724	62.8504	0.0173	0.5573

**Figure 2.**
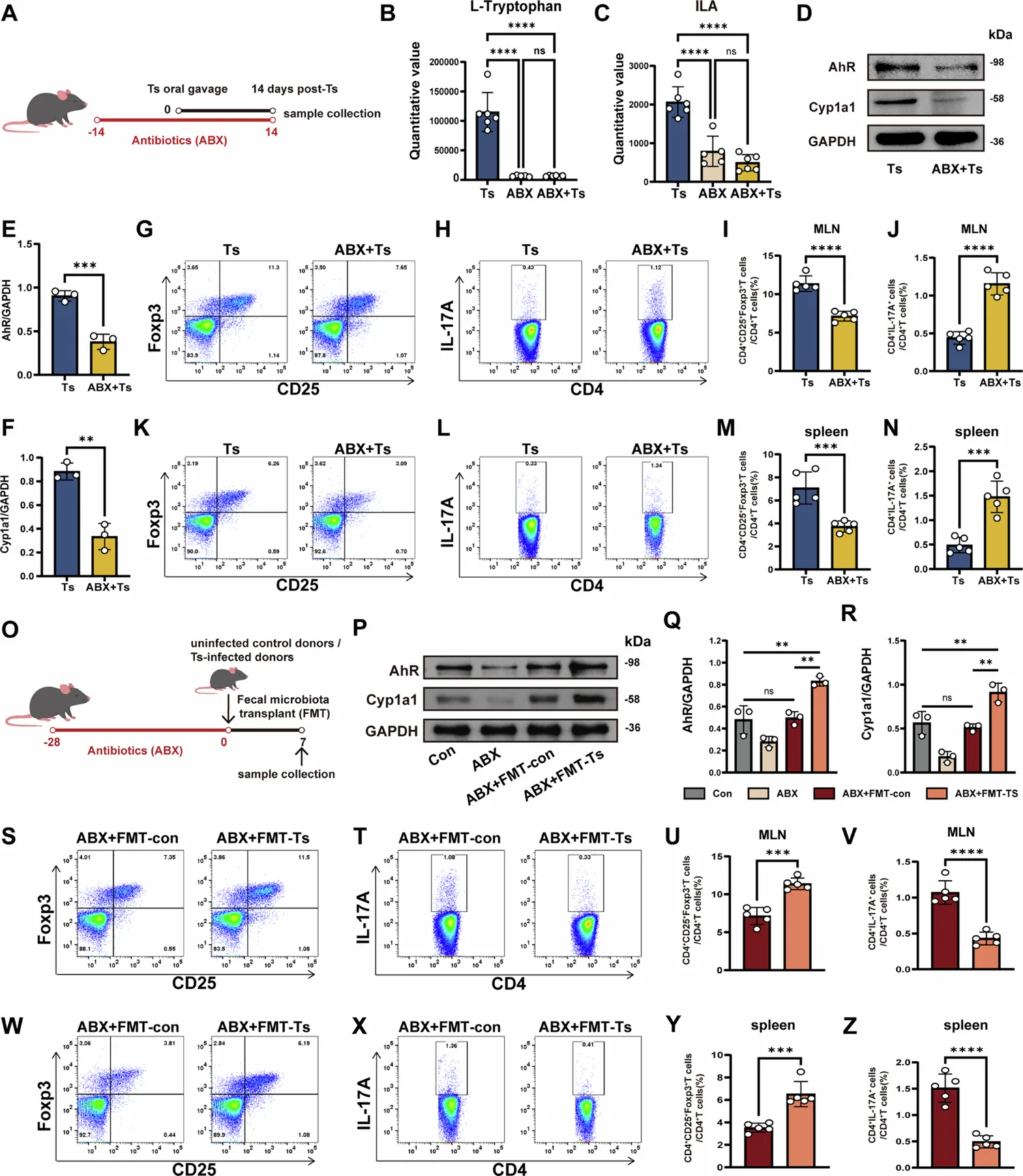
Ts-driven immunomodulation is mediated by the gut microbiota. (A–N) Antibiotic treatment abolishes Ts-induced AhR activation and immunomodulation. (A) Schematic of the antibiotic (ABX) treatment experiment (ampicillin 0.5 g/L, neomycin 0.5 g/L, metronidazole 0.5 g/L, vancomycin 0.25 g/L). (B, C) Fecal levels of L-Tryptophan and ILA (*n* = 6). (D–F) Western blot analysis of AhR and Cyp1a1 protein expression in the duodenum (*n* = 3). (G–N) Flow cytometric analysis of Treg (CD4^+^CD25^+^Foxp3^+^) and Th17 (CD4^+^IL-17A^+^) cells in MLNs (G to J) and spleen (K to N) (*n* = 5). (O–Z) Fecal preparations from Ts-infected donors (250 TsML) recapitulate AhR activation and immunomodulation. (O) Schematic of the FMT experiment. (P–R) Western blot analysis of AhR and Cyp1a1 protein expression in the duodenum (*n* = 3). (S–Z) Flow cytometric analysis of Treg (CD4^+^CD25^+^Foxp3^+^) and Th17 (CD4^+^IL-17A^+^) cells in MLNs (S to V) and spleen (W to Z) (*n* = 5). Data are representative of three independent experiments. Data are presented as mean ± SD and were analyzed by Student's *t*-test (E, F, I, J, M, N, U, V, Y and Z) or one-way ANOVA with Tukey's test (B, C, Q and R). ns, no statistical significance, **p < 0.01, ***p < 0.001, ****p < 0.0001.

### Ts infection enriched *L. murinus* and linked its expansion to an ILA-rich metabolic state

To identify the microbial taxa responsible for the observed metabolic changes, we performed metagenomic sequencing of fecal samples. The results showed that, compared to the control group, there were no significant differences in richness indices (Chao1 and observed species) or diversity indices (Simpson and Shannon) in the gut microbiota of Ts-infected mice (Figure S6A). However, Venn diagram analysis revealed a clear distinction in gut microbial composition between the different groups (Figure S6B). Principal coordinate analysis (PCoA) based on taxonomic abundance profiles revealed a significant separation between control and Ts-infected mice (Figure 3A). At the phylum level, the infected group showed an increase in the abundance of *Bacillota* and *Verrucomicrobia*. At the family level, gut microbiota remodeling in the infected group was characterized by elevated abundances of *Akkermansiaceae*, *Bacteroidaceae*, and *Lactobacillaceae* (Figure S6C). At the genus level, we observed an increase in the abundance of *Ligilactobacillus*, *Lactobacillus*, and *Akkermansia* in the infected group (Figure 3B). We performed Linear Discriminant Analysis Effect Size (LEfSe) with an LDA score threshold > 3. Notably, *L. murinus* showed the largest LDA effect size among taxa enriched in Ts-infected mice, nominating it as a candidate microbial contributor to the altered tryptophan-metabolic profile (Figure 3C and D). These analyses prioritized *L. murinus* for further investigation. We next integrated the taxonomic and metabolomic datasets to determine which tryptophan metabolite was linked to this microbial candidate. Spearman correlation analysis revealed a strong positive association between the abundance of *Ligilactobacillus* and fecal ILA levels in Ts-infected mice (Figure 3E), indicating that Ts-induced alterations in the gut microbiota contribute to indole metabolism. Combined with the marked increase in fecal and serum ILA after *T. spiralis* infection (Figure 1; Figure S2), this association prioritized ILA for experimental validation. In addition, Wilck et al. demonstrated that germ-free mice mono-colonized with *L. murinus* exhibited increased fecal tryptophan metabolites (ILA, IAld, IAA) versus control germ-free mice.^41^ Consistent with this observation, analysis of *L. murinus* culture supernatants showed a marked increase in ILA relative to control medium (681.2540 versus 13.9681 ng/mL; ~48.8-fold, *p* = 0.0022), accompanied by a significant reduction in tryptophan (62,129.5 versus 68,354.6 ng/mL; *p* = 0.0022) (Figure 3F and G; Table 4). These data demonstrate robust net ILA accumulation during *L. murinus* growth together with significant tryptophan consumption, supporting *L. murinus* as a functional contributor to the ILA-rich metabolic state. Together with its selective enrichment after *T. spiralis* infection, these findings connect *L. murinus* expansion to the infection-associated increase in ILA.

**Table 4.** Tryptophan metabolite levels in the culture supernatant of *L. murinus.*

Name	HMDB	Control(ng/mL)	*L. murinus*(ng/mL)	*p*-value	FC
2-Aminobenzoic acid	HMDB0001123	519.4168	600.4996	0.0022	1.1561
3-Hydroxyanthranilic acid	HMDB0001476	81.0234	68.1219	0.0087	0.8408
3-Hydroxykynurenine	HMDB0000732	40.0657	52.5204	0.0022	1.3109
Indoxylsulfate	HMDB0000682	1.0472	0.9875	0.3939	0.9430
Hydroxytryptophan	HMDB0000472	225.3724	235.7066	0.2403	1.0459
β-Indole-3-acetic acid	HMDB0000197	34.9758	34.2073	0.6991	0.9780
3-Indoleacetonitrile	HMDB0006524	0.3109	0.2565	0.3776	0.8250
Indole-3-acetamide	HMDB0029739	49.2694	53.7846	0.5887	1.0916
Indole-3-carbaldehyde	HMDB0029737	134.8311	140.7461	0.0649	1.0439
Tryptophol	HMDB0001855	71.8135	83.1967	0.0022	1.1585
Indole-3-lactic acid	HMDB0000671	13.9681	681.2540	0.0022	48.7723
Indolylpropionic acid	HMDB0002302	0.9261	0.5693	0.2403	0.6147
Kynurenic acid	HMDB0000715	28.6694	32.3318	0.0022	1.1277
L-kynurenine	HMDB0000684	48.6570	64.2935	0.0022	1.3214
L-tryptophan	HMDB0000929	68354.6000	62129.5000	0.0022	0.9089
Nicotinic acid	HMDB0001488	2183.3520	10304.5400	0.0022	4.7196
N-Formylanthranilic acid	HMDB0004089	26.8015	27.1009	0.9372	1.0112
N-formylkynurenine	HMDB0001200	49.8586	56.5956	0.0022	1.1351
Picolinic acid	HMDB0002243	1684.1120	6875.8600	0.0022	4.0828
Tryptamine	HMDB0004370	42.6252	41.9523	0.3939	0.9842
Xanthurenic acid	HMDB0000881	30.9173	29.7177	0.1797	0.9612

**Figure 3.**
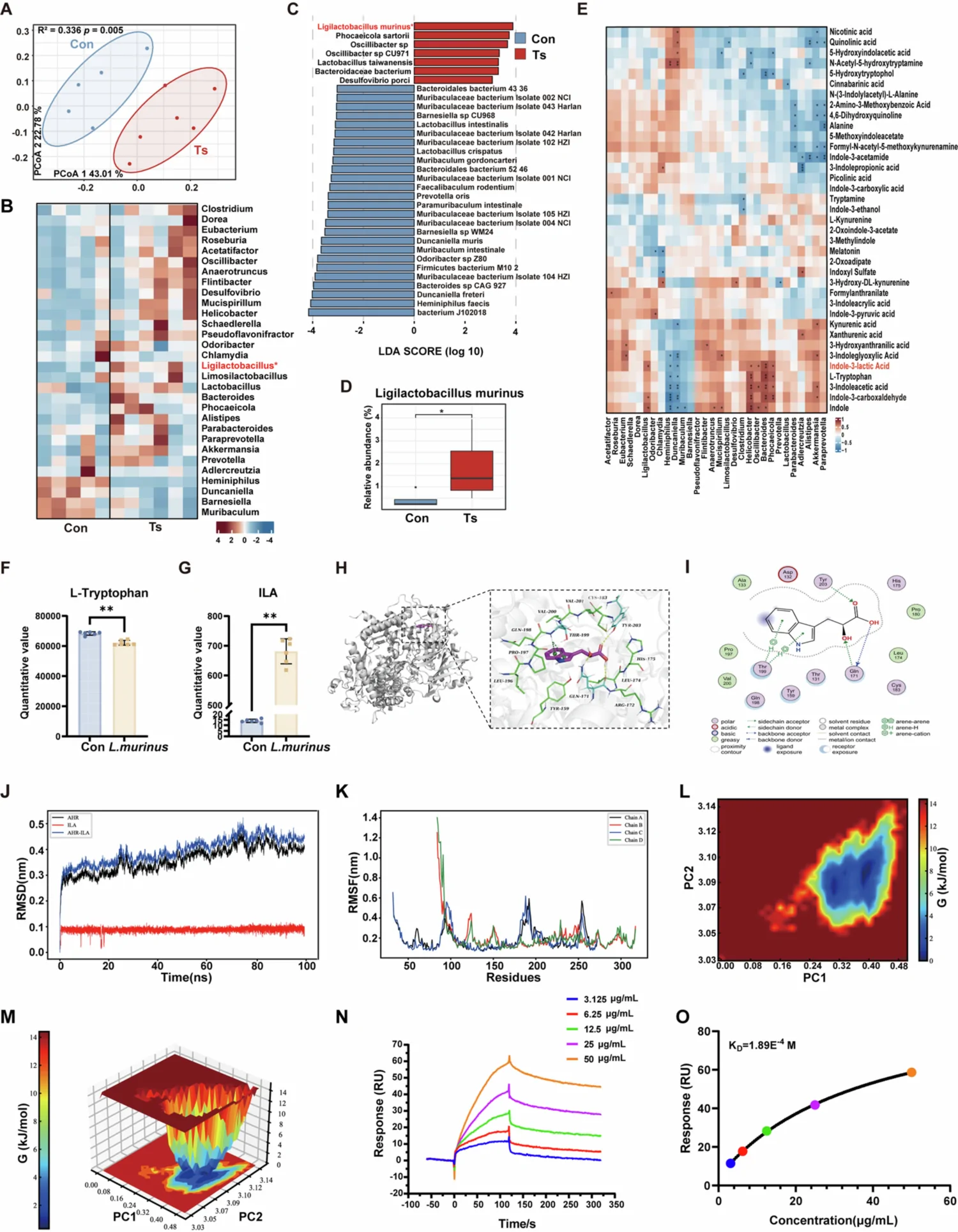
Ts infection enriches *L. murinus* and links its expansion to an ILA-rich metabolic state. (A) PCoA plot showing distinct gut microbial composition in Ts-infected mice. (B) Heatmap of relative bacterial abundance at the genus level. (C) LEfSe analysis identifying enriched bacterial taxa (LDA score > 3, Kruskal-Wallis test). (D) Relative abundance of *L. murinus* in control and Ts-infected mice. (E) Spearman correlation matrix linking bacterial taxa with tryptophan metabolites. (F, G) Targeted metabolomic analysis showing robust ILA accumulation and concomitant tryptophan depletion during *L. murinus* culture (*n* = 6). (H–M) Molecular docking and dynamics simulations demonstrating stable binding of ILA to the human AhR ligand-binding pocket. (J, K) Root-mean-square deviation (RMSD) and fluctuation (RMSF) plots indicating system stability. (N, O) Surface Plasmon Resonance (SPR) analysis quantifying the direct binding affinity between ILA and recombinant human AhR. The sensorogram shows binding responses at graded concentrations. Note: Concentrations were prepared in μg/mL (3.125, 6.25, 12.5, 25, 50). Steady-state affinity fitting yields a calculated K_D_ of 1.89E^−4^M. Data are presented as mean ± SD. Statistical analysis for metagenomic data (C) was performed using the Kruskal–Wallis test. Comparisons in (D, F, and G) were analyzed using an unpaired Student's *t*-test. *p < 0.05, **p < 0.01.

To elucidate the molecular basis of the interaction between ILA and AhR, we first employed molecular docking to simulate ILA binding to human AhR.^19^ The computational model predicted that ILA occupies a hydrophobic pocket within human AhR, with key residues Thr199, Gln171, and Tyr203 contributing to the predicted binding interactions (Figure 3H and I). The calculated binding energy of −6.7 kcal·mol^−1^ suggested a high-fidelity interaction, supporting ILA as a direct, physiologically relevant but relatively low-affinity microbial ligand for AhR. To further evaluate the dynamic stability of this interaction under physiological conditions, we performed 100 ns molecular dynamics (MD) simulations. The root-mean-square deviation (RMSD) and fluctuation (RMSF) profiles reached convergence quickly and remained stable throughout the trajectory, indicating that ILA binding induces a compact and robust conformation of the AhR molecule (Figure 3J and K). Importantly, we constructed the Free Energy Landscape (FEL) based on the MD trajectories to explore the thermodynamic properties of the complex. The FEL analysis identified a distinct, well-defined energy minimum (deep blue basin), confirming that ILA binding stabilizes AhR in a thermodynamically favored state, which is a hallmark of effective receptor activation (Figure 3L and M). We then performed Surface Plasmon Resonance (SPR) analysis using recombinant human AhR to quantify direct ILA–AhR binding and provide biophysical evidence of receptor engagement. ILA exhibited a dose-dependent binding response with a calculated equilibrium dissociation constant (K_D_) of 1.89E^−4^M (Figure 3N and O). Although this affinity is substantially weaker than that of high-affinity xenobiotic AhR ligands, it supports direct engagement of AhR by ILA within a concentration range that may be relevant for locally abundant microbial metabolites.

### *L. murinus* and ILA supplementation recapitulated AhR activation and Treg/Th17 modulation

Having identified *L. murinus* and its metabolite ILA as key mediators, we next tested whether their administration alone could recapitulate the immunomodulatory effects of Ts infection in antibiotic treated mice. We examined whether *L. murinus* supplementation affected intestinal adult worm burden during Ts infection. Mice were orally administered *L. murinus* continuously for 14 d after infection, and the intestinal adult worm burden was assessed at 14 dpi. Compared with the control group, *L. murinus* supplementation significantly increased the intestinal adult worm burden (Figure 4A). After antibiotic pretreatment, the experimental groups were supplemented with *L. murinus*. After continuous intervention for 7 d, relevant indicators in intestinal tissues were assessed. Compared to vehicle controls, supplementation with *L. murinus* significantly increased both mRNA and protein expression of AhR and Cyp1a1 in the intestine (Figure 4B–D; Figure S7A and B). Additionally, mRNA expression of Foxp3 and TGF-β was significantly upregulated in the intervention groups, while expression of IL-17A was markedly suppressed compared to the control group (Figure S7C). Flow cytometric analysis further demonstrated that *L. murinus* supplementation significantly increased the proportion of CD4⁺CD25⁺Foxp3⁺ Treg cells while reducing the proportion of CD4⁺IL-17A⁺ Th17 cells in the mesenteric lymph nodes (MLNs) (Figure 4E–H). Similar results were also observed in the flow cytometric analysis of the spleen, where *L. murinus* markedly promoted Treg cell expansion and suppressed Th17 cell differentiation (Figure 4I–L). These findings indicate that *L. murinus* activates the AhR signaling pathway and shifts the Treg/Th17 balance toward immune tolerance. To test whether exogenous ILA was sufficient to reproduce the infection-associated immunoregulatory program, antibiotic-treated mice received the defined pharmacological supplementation regimen (20 mg/kg/d for 7 d). Intestinal adult worm burden analysis showed that, compared with the Ts-infected group, ILA supplementation also significantly increased the small intestinal adult worm burden at 14 dpi (Figure 4M). Compared with vehicle controls, ILA significantly enhanced both the mRNA and protein expression levels of AhR and Cyp1a1 in intestinal tissues (Figure 4N–P; Figure S7D andE). qPCR analysis further confirmed that ILA significantly upregulated the mRNA expression of Foxp3 and TGF-β while suppressing the expression of IL-17A (Figure S7F). Flow cytometric analysis showed that ILA significantly increased the proportion of CD4⁺CD25⁺Foxp3⁺ Treg cells and decreased the proportion of CD4⁺IL-17A⁺ Th17 cells in both the MLNs and spleen (Figure 4Q–X). Overall, gut microbiota-derived indole metabolites activate the AhR pathway, increasing Treg frequencies while reducing Th17 frequencies. Previous studies have shown that Treg-mediated immune tolerance facilitates parasite persistence by suppressing host anti-parasitic immunity,^42^ which may explain the increased intestinal adult worm burden following *L. murinus* and ILA supplementation. *In vitro* stimulation of intestinal organoids with *L. murinus* or ILA significantly upregulated mRNA expression levels of AhR and Cyp1a1 in a dose-dependent manner, as determined by qPCR analysis (Figure S7G–L). In intestinal organoids, *L. murinus* or ILA increased AhR and Cyp1a1 expression, and this induction was attenuated by AhR antagonist treatment, indicating that the *L. murinus*–ILA axis can activate AhR signaling in the intestinal epithelial compartment (Figure 4Y).

**Figure 4.**
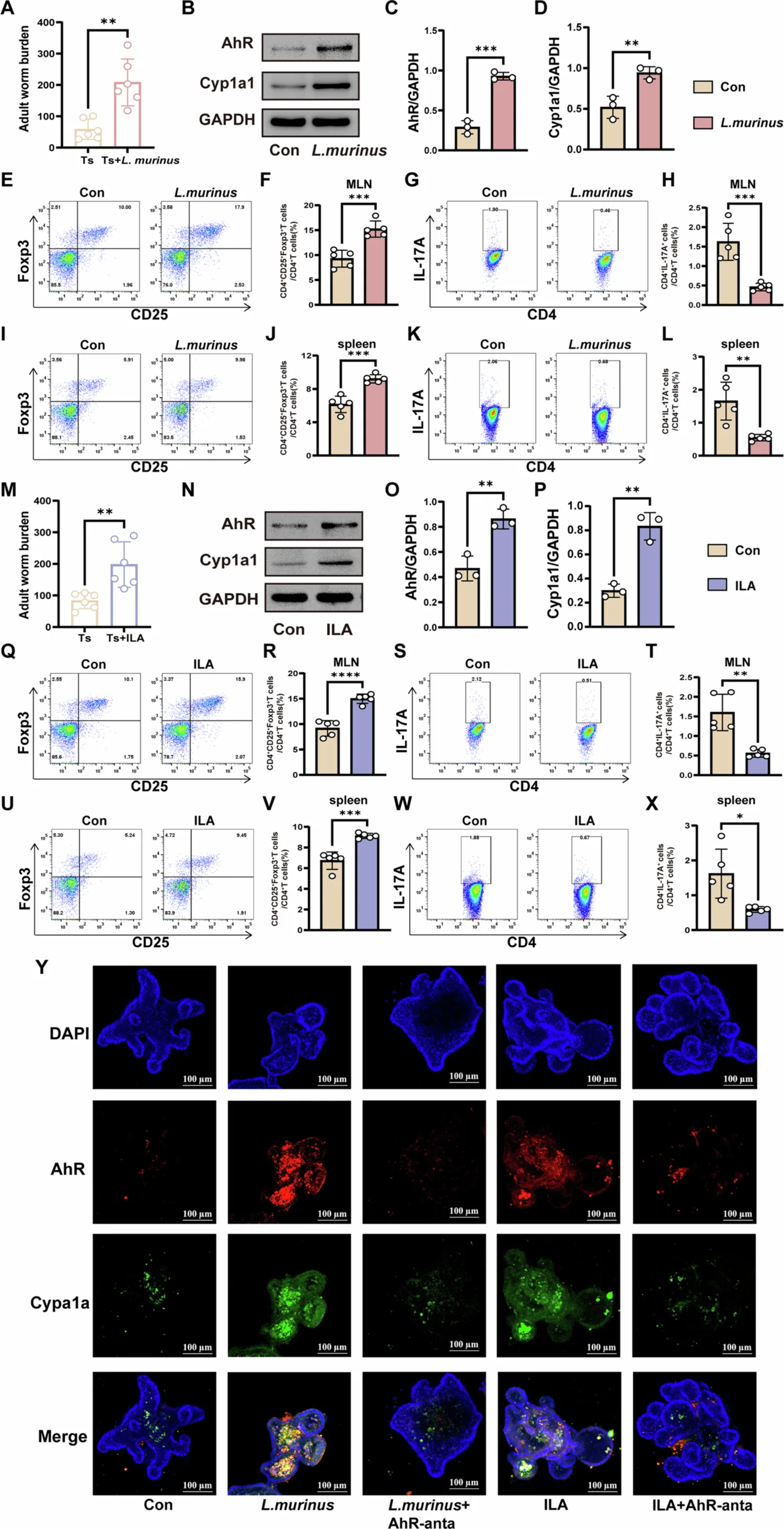
Supplementation with *L. murinus* or ILA recapitulates AhR activation and immunomodulation. (A) Mice were orally infected with 500 TsML, followed by oral administration of vehicle control or *L. murinus* (1 × 10^9^ CFU/mL) from 1 to 14 dpi, and at 14 dpi, adult worms were recovered from each group to assess the burden of *T. spiralis* (*n* = 6). (B–D) *in vivo* effects in antibiotic-treated mice. Western blot analysis and quantification of AhR and Cyp1a1 protein levels in the duodenum after supplementation with vehicle, *L. murinus* (1 × 10^9^ CFU/mL) for 7 d (*n* = 3). (E–L) Flow cytometric analysis of Treg (CD4^+^CD25^+^Foxp3^+^) and Th17 (CD4^+^IL-17A^+^) cells in MLNs (E, F, G and H) and spleen (I, J, K and L) after supplementation with vehicle, *L. murinus* (1 × 10^9^ CFU/mL) for 7 d after 21 d of antibiotic pretreatment (*n* = 5). (M) Mice were orally infected with 500 TsML, followed by oral administration of vehicle control or ILA (20 mg/kg) from 1 to 14 dpi, and at 14 dpi, adult worms were recovered from each group to assess the burden of *T. spiralis* (*n* = 6). (N–P) *in vivo* effects in antibiotic-treated mice. (N–P) Western blot analysis and quantification of AhR and Cyp1a1 protein levels in the duodenum after supplementation with vehicle, ILA (20 mg/kg) for 7 d (*n* = 3). (Q–X) Flow cytometric analysis of Treg (CD4^+^CD25^+^Foxp3^+^) and Th17 (CD4^+^IL-17A^+^) cells in MLNs (Q, R, S and T) and spleen (U, V, W and X) after supplementation with vehicle, ILA (20 mg/kg) for 7 d after 21 d of antibiotic pretreatment (*n* = 5). (Y) Representative immunofluorescence images confirming increased AhR and Cyp1a1 protein in organoids treated with *L. murinus* (1 × 10^6^ CFU/mL) or ILA (2 mM) for 48 h. Data are representative of three independent experiments. Data are presented as mean ± SD and were analyzed by Student's *t*-test (A, C, D, F, H, J, L, M, O, P, R, T, V, and X). *p < 0.05, **p < 0.01, ***p < 0.001, ****p < 0.0001.

### Ts-*L. murinus*-ILA axis conferred AhR-dependent protection against LPS-induced cytokine storm

To determine whether the Ts-*L. murinus*-ILA axis confers functional protection against hyperinflammation, we used a murine model of LPS-induced cytokine storm. Ts infection markedly attenuated this inflammatory syndrome, as reflected by reduced LPS-induced lung pathology and inflammatory cell infiltration (Figure 5A and B). Moreover, it lowered serum levels of the pro-inflammatory cytokines tumor necrosis factor-α (TNF-α) and interferon-γ (IFN-γ), increased the Treg-associated anti-inflammatory cytokines IL-10 and TGF-β, and decreased the Th17-associated cytokine IL-17A (Figure 5C–G). This protection was lost following pretreatment with CH223191, a pharmacological AhR antagonist.^43^ In line with our hypothesis, supplementation with either *L. murinus* or ILA recapitulated the protective effects of Ts infection, significantly reducing lung histopathology scores and inflammatory infiltration in the LPS-induced cytokine storm model (Figure 5H and I). Serum levels of TNF-α, IFN-γ, and IL-17A were decreased, while IL-10 and TGF-β levels were elevated, closely mirroring the immunoregulatory profile induced by Ts infection (Figure 5J- N). Pretreatment with CH223191 similarly abolished the protective effects of *L. murinus* and ILA. To determine whether these pharmacological phenotypes were recapitulated genetically, we examined the same protective endpoints in AhR-KO mice. In AhR-KO mice, Ts infection, *L. murinus*, and ILA failed to reduce LPS-induced lung pathology or reproduce the protective cytokine profile observed in WT mice (Figure 5O–U). Thus, pharmacological AhR inhibition and genetic AhR deletion produced concordant loss-of-protection phenotypes across lung pathology and cytokine readouts, providing convergent evidence that AhR is required for protection mediated by the Ts–*L. murinus*–ILA–AhR axis.

**Figure 5.**
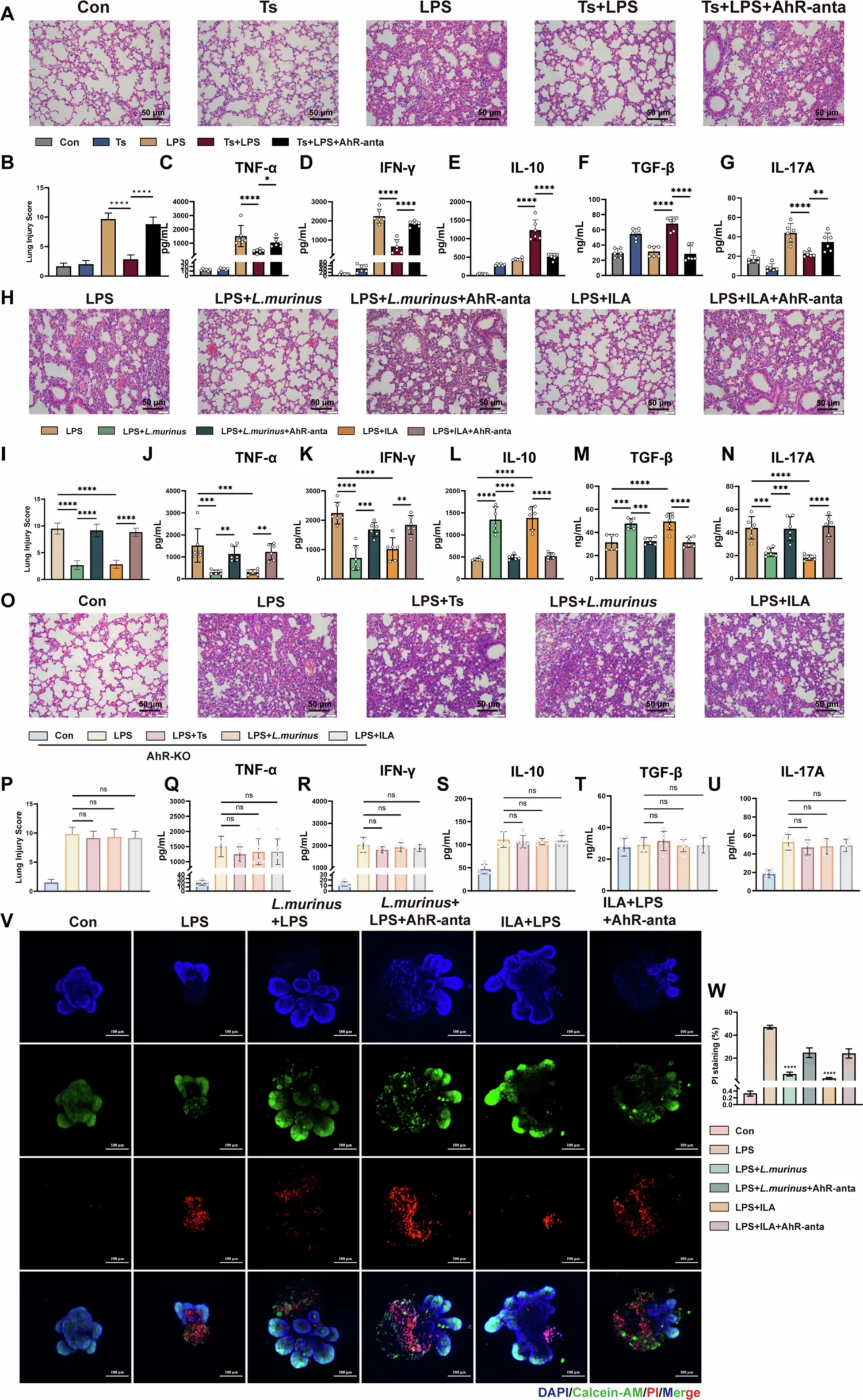
The Ts-*L. murinus*-ILA axis protects against LPS-induced cytokine storm through AhR. (A–G) Ts infection protects wild-type (WT) mice from LPS (8 mg/kg)-induced cytokine storm, an effect reversed by the AhR antagonist CH223191 (10 mg/kg). (A) Representative H&E staining of lung sections. (B) Lung histopathological scores. (C–G) Serum cytokine levels. (H–N) Supplementation with *L. murinus* (1 × 10^9^ CFU/mL) or ILA (20 mg/kg) mimics the protective effects in WT mice. (H) Representative lung H&E staining. (I) Lung scores. (J–N) Serum cytokine levels. (O–U) The protective effects of Ts infection, *L. murinus*, or ILA are completely abolished in AhR-knockout (AhR-KO) mice. (O) Representative lung H&E staining. (P) Lung scores. (Q–U) Serum cytokine levels. (V, W) *L. murinus* and ILA protect intestinal organoids from LPS-induced damage *in vitro*. (V) Data are representative of three independent experiments. Representative images of organoids with PI staining indicating cell death. (W) Quantification of damaged organoids. *n* = 6 for *in vivo* experiments and *n* = 3 for *in vitro* experiments. Scale bars, 100 μm. Data are presented as mean ± SD. Statistical analysis was performed using one-way ANOVA with Tukey's multiple comparison test (B, C, D, E, F, G, I, J, K, L, M, N, P, Q, R, S, T, U and W). ns, no statistical significance, *p < 0.05, ***p < 0.001, ****p < 0.0001.

*In vitro*, LPS treatment increased propidium iodide (PI)-positive cell death and reduced organoid viability, whereas *L. murinus* or ILA improved viability in an AhR-dependent manner (Figure 5V and W). QPCR and ELISA results further demonstrated decreased expression of pro-inflammatory cytokines TNF-α and IFN-γ (Figure S8A–D). Notably, this protective effect was abolished by CH223191 treatment. Collectively, these findings indicate that Ts exerts anti-inflammatory and protective effects by activating the AhR pathway through the *L. murinus*-ILA axis, thereby modulating the Treg/Th17 cells balance.

### ILA improves disease outcome after lethal SARS-CoV-2 challenge

Cytokine storm represents a significant cause of morbidity and mortality in patients with COVID-19.^44^ ^,^ ^45^ We further evaluated whether ILA could alter disease outcome after SARS-CoV-2 infection (Figure 6A). ILA treatment markedly improved disease outcomes in SARS-CoV-2-infected K18-hACE2 mice. While SARS-CoV-2 infection caused rapid mortality, with all mice succumbing by 9 dpi, oral administration of ILA substantially increased survival, with approximately 60% of mice surviving to 14 dpi (Figure 6B). Consistently, infected mice showed progressive body-weight loss, whereas ILA-treated mice exhibited significantly attenuated weight loss and partial recovery during the later phase of infection (Figure 6C). At 6 dpi, pulmonary viral load was comparable between SARS-CoV-2-infected mice with or without ILA treatment, indicating that ILA did not significantly reduce lung viral burden (Figure 6D). However, ILA markedly reshaped the pulmonary cytokine response. SARS-CoV-2 infection increased lung TNF-α, IFN-γ, and IL-17A levels, whereas ILA treatment significantly reduced these pro-inflammatory cytokines (Figure 6E, F and I). In contrast, ILA strongly enhanced IL-10 production and moderately increased TGF-β levels in infected lungs (Figure 6G and H). The improvements were observed in the histopathology in the lungs of the mice treated with ILA (Figure 6J and K). Together, these results indicate that ILA protects SARS-CoV-2-infected K18-hACE2 mice without directly suppressing viral replication, but instead by limiting excessive pulmonary inflammation and promoting a regulatory cytokine profile.

**Figure 6.**
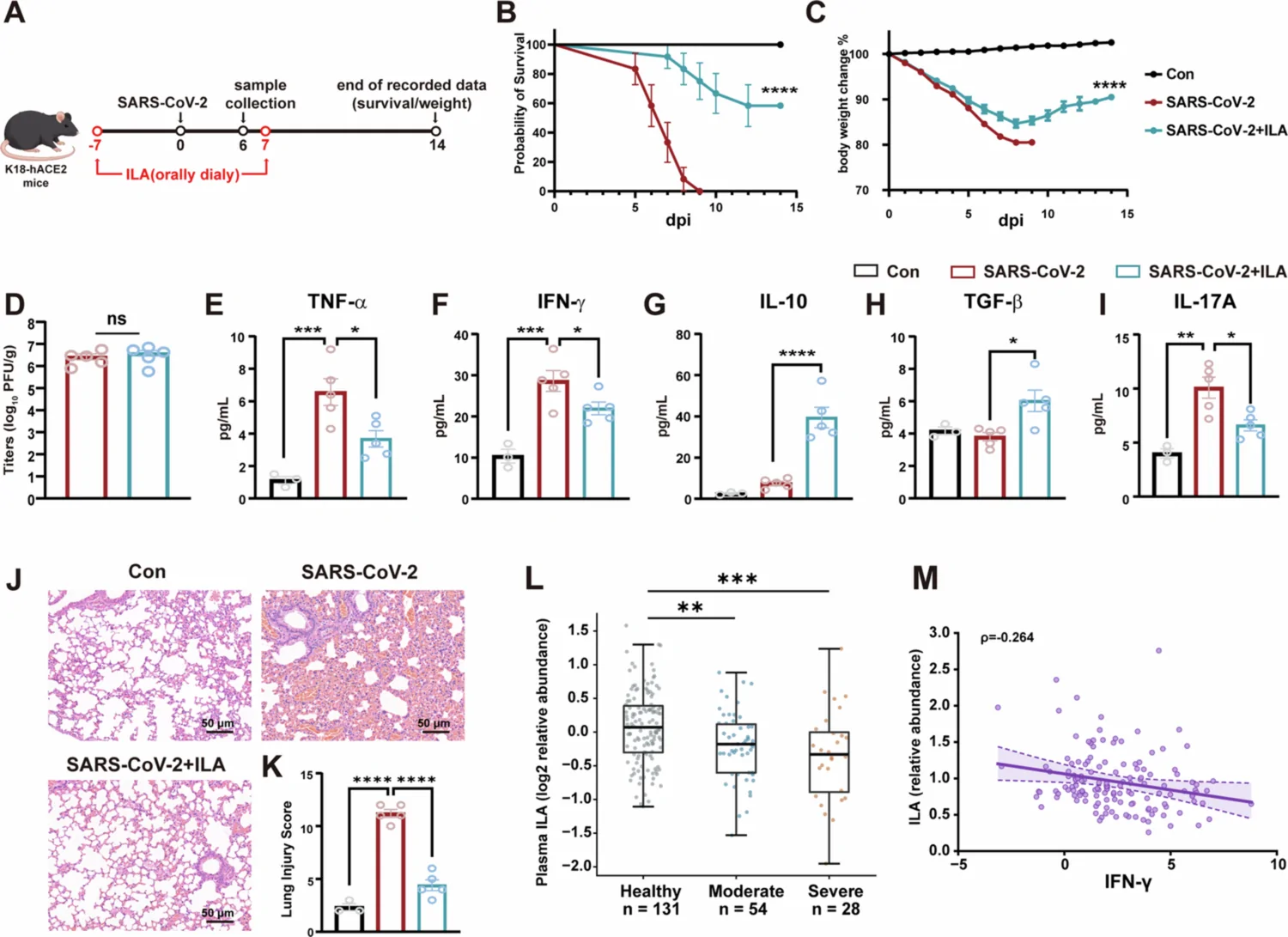
ILA improves survival after SARS-CoV-2 infection in mice and is associated with circulating IFN-γ in COVID-19 patients. (A) Experimental design to determine whether ILA (20 mg/kg) protected against SARS-CoV-2 infection in 5- to 6-week-old K18-hACE2 mice. (B) Survival of ILA-treated K18-hACE2 mice after SARS-CoV-2 infection (*n* = 12). (C) Percent changes of body weights of ILA-treated K18-hACE2 mice after SARS-CoV-2 infection (*n* = 12). (D) Lung viral load of mice after SARS-CoV-2 infection (*n* = 5). (E–I) Serum cytokine levels (*n* = 3–5). (J) Representative H&E staining of lung sections (*n* = 3–5). (K) Lung histopathological scores (*n* = 3–5). (L) Plasma ILA, displayed as log2 relative abundance, in 131 independent healthy donors and verified T1 draws from 54 moderate (WOS 3–4) and 28 severe (WOS 5–7) patients. Each point represents one person. Boxplots show the median and interquartile range, with whiskers extending to observations within 1.5 times the interquartile range. (M) Paired plasma ILA and IFN-γ measurements from 84 patients (142 draws). The x-axis shows IFN-γ Olink NPX; the y-axis shows source ILA relative abundance. The line is the pooled, unadjusted ordinary least-squares mean fit; shading denotes its pointwise 95% confidence band from 2000 patient-cluster bootstrap refits, preserving repeated draws within each resampled patient. Descriptive Spearman *ρ* = −0.264 (patient-cluster bootstrap 95% CI −0.429 to −0.085). Adjusted GEE estimates are reported in the Supplementary statistical table. Human data were reconstructed from the published cohort of Su et al.^36^; severity was classified at each blood draw. Statistical analysis was performed using the survival curve comparison (log-rank [Mantel-Cox] test) (B), Student's *t*-test (D) and one-way ANOVA with Tukey's multiple comparison test (C, E, F, G, H, I and K). ns, no statistical significance, *p < 0.05, **p < 0.01, ***p < 0.001, ****p < 0.0001. Panel L used Welch tests on log2 ILA with Holm correction. Panel M uses patient-cluster bootstrap intervals to account for repeated sampling. Exact tests, adjusted GEE models and strict T1-only analyses appear in the Supplementary statistical table.

We reanalyzed the plasma multi-omics cohort of Su et al.^36^ Figure 6L includes 131 healthy donors and verified T1 samples from 54 moderate and 28 severe patients. Mean log2 ILA relative abundance was lower in both patient groups than in controls (Holm-adjusted *p* = 0.00347 and 0.00828, respectively); the moderate–severe comparison gave *p* = 0.335. The same-draw ILA–IFN-γ analysis included 142 observations from 84 patients after excluding six values without observed source ILA. In the common complete-case population (82 patients, 140 draws), M0 gave β = −1.124, 95% CI −1.655 to −0.592, p = 3.39e-05; M1 gave β = −1.187, 95% CI −1.719 to −0.654, p = 1.27e-05; M2 gave β = −0.949, 95% CI −1.492 to −0.406, *p* = 0.00062; and M3 gave β = −0.830, 95% CI −1.409 to −0.251, *p* = 0.00495. Coefficients represent IFN-γ NPX differences per doubling of ILA relative abundance. Descriptive pooled Spearman *ρ* was −0.264 (patient-cluster bootstrap 95% CI −0.429 to −0.085; Figure 6M). Among 78 verified T1 complete cases, M2 gave β = −0.809, 95% CI −1.674 to 0.056, *p* = 0.0664 and M3 gave β = −0.689, 95% CI −1.633 to 0.256, *p* = 0.15. The first-draw estimates were directionally consistent, with confidence intervals including zero. Patient-clustered models supported an inverse association after demographic and clinical adjustment.

## Discussion

Persistent infection exposes a fundamental constraint on immunity: host survival depends on limiting tissue damage even when pathogen elimination is incomplete. Here, we identify a multi-kingdom mechanism through which *T. spiralis* shifts this balance. Infection reorganized the intestinal ecosystem toward expansion of the ILA-associated commensal *L. murinus*, increased the intestinal ILA pool, and activated AhR-dependent regulatory immunity. The causal architecture was supported at each level: antibiotic perturbation interrupted ILA production and immune remodeling; transfer of the infection-conditioned fecal preparations restored the phenotype; *L. murinus* or ILA reproduced the major metabolic and immunological outputs; and pharmacologic or genetic disruption of AhR extinguished protection. These convergent perturbations establish the *T. spiralis*–*L. murinus*–ILA–AhR axis as a mechanistically connected pathway. Helminth-induced tolerance therefore emerges from coordinated activity across parasite, microbiota, and host, extending the immunoregulatory repertoire of helminths beyond parasite-encoded effectors.

This framework advances helminth–microbiota biology from community association to ecological mechanism. Previous studies established that helminths reshape intestinal microbial communities and that the microbiota can contribute to suppression of inflammatory disease.^7^ ^,^ ^8^ Our data identify the functional unit that carries this information: a specialized microbial metabolic activity linked to a defined host sensor and a disease-relevant outcome. Notably, *T. spiralis* infection altered community structure without a broad increase in alpha diversity, indicating that immune consequences can arise from selective functional reorganization rather than global gains in richness. The convergence of species-level *L. murinus* enrichment, the genus-level positive association between *Ligilactobacillus* and fecal ILA, robust ILA accumulation with concomitant tryptophan consumption in culture, and its ability to reproduce the downstream AhR–Treg/Th17 program *in vivo* identifies *L. murinus* as a functionally supported contributor to the infection-associated ILA pool. Other infection-enriched taxa may provide additional ILA-generating capacity through parallel or community-level metabolic interactions, indicating that the regulatory chemistry can emerge from both species-level and ecosystem-level organization. This observation aligns with the view that microbial metabolites operate as multi-kingdom intermediates and suggests that the immunological phenotype of helminth infection is encoded in the metabolic state of the ecosystem it constructs.^14^ Taxonomic composition remains important, yet the more transferable biological principle is the capacity of a community to generate regulatory chemistry.

An upstream question is how *T. spiralis* remodels the intestinal microbial ecosystem and creates a niche that favors *L. murinus*. Our data, together with established mechanisms of helminth-induced mucosal remodeling, support a model of convergent ecological selection involving mucosal remodeling, antimicrobial filtering, and altered nutrient availability. *T. spiralis* elicits a strong type-2 mucosal response characterized by IL-13-dependent goblet-cell expansion and altered mucin production. These changes remodel the physical and nutritional landscape of the intestinal lumen and therefore directly alter the ecological conditions experienced by resident bacteria.^46^ ^,^ ^47^ Consistent with this framework, longitudinal analysis of *T. spiralis* infection has demonstrated stage-dependent restructuring of the intestinal microbiota and metabolome, including enrichment of lactobacilli together with marked changes in amino-acid and bile-acid metabolism.^12^ Helminth-induced innate antimicrobial programs provide a complementary mechanism for selective ecological filtering. Zou et al. demonstrated that helminth infection expands CD11b⁺CD206⁺ macrophages that produce CRAMP, which preferentially suppresses Gram-negative competitors and thereby increases the ecological fitness of intestinal lactobacilli. Importantly, supporting observations were also reported in a *T. spiralis*-mouse system, providing direct relevance of this antimicrobial-selection principle to *T. spiralis* infection.^48^ Our metabolomic data further identify a complementary metabolic component of this niche: *T. spiralis* infection increased fecal tryptophan from 61,350.36 to 115,036.50 ng/g (~1.87-fold), and independent intestinal ecosystem experiments have shown that increased tryptophan availability can increase *Lactobacillus* abundance and enhance microbial production of tryptophan-derived metabolites.^49^ We therefore interpret *T. spiralis*-induced microbiota remodeling as host-mediated ecological engineering: parasite-induced type-2 mucosal remodeling changes the mucus and epithelial habitat; innate antimicrobial programs alter interbacterial competition; and infection-associated metabolic changes alter substrate availability. These processes jointly create a selective niche in which *L. murinus* and related lactobacilli gain ecological fitness. Once enriched, *L. murinus* can translate this remodeled niche into the ILA-rich metabolic program characterized experimentally in our study.

ILA provides a plausible molecular language for this interkingdom communication. Microbial tryptophan catabolites are diffusible signals that couple nutrient availability and bacterial metabolism to epithelial and immune programs.^15^ ^,^ ^38^ ILA bound directly to AhR and induced canonical AhR-responsive outputs in intestinal tissue and organoids. Its moderate binding affinity is compatible with the behavior of a physiological microbial ligand whose activity may depend on sustained local production, tissue exposure, and ligand competition, rather than the persistent high-amplitude activation associated with xenobiotic agonists. AhR is a context-sensitive interpreter of environmental signals, with transcriptional outcomes determined by ligand structure, responding cell type, tissue compartment, and inflammatory state.^17^ In the present setting, AhR activation was coupled to increased Foxp3, IL-10, TGF-β,and Treg abundance and to reduced IL-17A and Th17 responses. This immune configuration is consistent with prior evidence that *L. murinus* can restrain the Th17 axis, while our findings define ILA and AhR as the molecular bridge. The organoid data further indicate that the epithelial compartment can directly sense this pathway, raising the possibility that epithelial AhR initiates secondary signals that organize systemic lymphocyte responses. Cell-restricted genetic studies will be required to resolve the relative contributions of epithelial, myeloid, and lymphoid AhR.

The most consequential functional observation is the separation of survival from pathogen control. Oral ILA improved survival in lethal SARS-CoV-2 infection and reduced pulmonary injury while leaving lung viral burden unchanged. This decoupling defines a disease-tolerance phenotype: the host maintained function by constraining immune-mediated damage without measurably enhancing viral clearance. The same axis protected against endotoxin-driven hyperinflammation, and its benefit disappeared when AhR was blocked or deleted. Convergence across mechanistically distinct inflammatory insults suggests that the pathway acts on a shared host-damage program rather than on a pathogen-specific process. This property has broad therapeutic relevance because severe infectious disease often reflects the combined effects of pathogen burden and dysregulated host inflammation. In the human cohort, the inverse association between circulating ILA and IFN-γ persisted after adjustment for demographic and available clinical covariates, providing clinical evidence consistent with the experimental findings.^36^ Disturbed microbial tryptophan metabolism has also been linked to acute and post-acute inflammatory outcomes after SARS-CoV-2 infection.^50^ Together, these experimental and observational findings support the clinical relevance of the ILA–inflammation relationship and motivate prospective studies of ILA abundance, microbial ILA-producing capacity and AhR-response signatures.

The increase in intestinal worm burden after *L. murinus* or ILA supplementation gives this circuit an evolutionary dimension. The regulatory state that protected host tissues also weakened anti-helminth pressure, thereby extending the parasite's opportunity for persistence. The pathway can therefore be viewed as a negotiated tolerance program with reciprocal fitness consequences: the parasite gains a more permissive niche, while the host gains protection from inflammatory injury. Regulatory T cells are established determinants of susceptibility and persistence during parasite infection, and the present findings place microbial metabolism upstream of this equilibrium.^42^ A metazoan parasite can thus stabilize long-term coexistence by altering the metabolic output of a commensal partner. This ecological trade-off may help explain how chronic helminth infection persists for prolonged periods without proportionate immunopathology. Reciprocally, these findings nominate the microbial ILA–AhR circuit as a candidate anti-helminth target: selectively reducing microbial ILA production or local ILA–AhR signaling is predicted to shift the host–parasite equilibrium toward stronger parasite control. This experimentally testable prediction extends the pathway from a mechanism of tolerance to a potential lever on parasite persistence.

The translational implication is a function-centered postbiotic strategy. *L. murinus* is adapted to the murine gut and is unlikely to serve as a universal human therapeutic organism; the conserved intervention target is ILA-generating capacity. Human-associated lactobacilli and bifidobacteria can produce ILA creating opportunities to identify human microbial producers, engineer defined consortia, or deliver the metabolite directly.^51^ ^,^ ^52^ Such approaches could capture a beneficial output of helminth exposure while avoiding the biological complexity and safety liabilities of live-parasite therapy. A tolerance-directed intervention may be particularly valuable as an adjunct to antiviral or antimicrobial treatment when pathogen-directed control is insufficient to prevent tissue injury. Clinical development will require precise control of dose, timing, and tissue exposure. The same regulatory program that limits cytokine-driven pathology may impair pathogen clearance, vaccine responses, or antitumor immunity in other settings, consistent with the ligand- and context-dependent biology of AhR.^19^ Therapeutic use should therefore be guided by disease stage, microbial and metabolic biomarkers, and the relative contribution of immunopathology to clinical outcome.

Broad-spectrum antibiotic treatment can influence host physiology independently of changes in the microbiota, and such microbiota-independent effects are an important consideration when antibiotic-treated animals are used to perturb the gut microbiota.^53^ ^,^ ^54^ In our study, this potential confounding was addressed by the experimental structure: recipients of control- and Ts-conditioned fecal preparations underwent the same antibiotic pretreatment, as did mice assigned to vehicle, *L. murinus*, or ILA supplementation. Under these shared antibiotic backgrounds, Ts-conditioned fecal preparations, *L. murinus*, and ILA selectively restored AhR/Cyp1a1 activation and Treg/Th17 remodeling relative to their matched controls. Thus, a generalized direct host effect of the antibiotic regimen is insufficient to explain the donor-dependent and supplementation-dependent restoration of the phenotype. Together with the phenotype established in conventionally colonized, non-antibiotic-treated Ts-infected mice and the concordant pharmacological and genetic AhR loss-of-function data, these convergent experiments support a causal microbiota-metabolite contribution to the Ts-induced regulatory program.

Importantly, antibiotic treatment did not measurably alter intestinal adult *T. spiralis* burden in the present study, providing an internal control against parasite-load differences as an explanation for the ABX-associated metabolic and immunological phenotypes. This observation is consistent with our previous study using the same four-antibiotic combination, in which antibiotic treatment did not significantly alter either intestinal adult worm burden at 7 dpi or muscle-larval burden at 35 dpi.^55^ Together, these concordant findings further support the interpretation that the altered metabolic and immunoregulatory phenotype after antibiotic treatment reflects disruption of the intestinal microbial ecosystem rather than a secondary consequence of altered parasite burden.

The strong AhR dependence observed here should be interpreted within the broader diversity of helminth immunoregulatory strategies. In *H. polygyrus*, prolonged microbiota abrogation leaves major effector, memory, and recall Th2 responses and primary-infection worm burdens largely intact,^56^ while the parasite secretes Hp-TGM, a TGF-β mimic that directly engages mammalian TGF-β receptors and potently induces Foxp3+Tregs.^57^ Yet germ-free infection reduces intestinal Foxp3+RORγt+regulatory T cells and parasite fecundity, indicating that direct parasite-derived and microbiota-supported regulation can coexist.^58^ *T. spiralis* likewise secretes products capable of conditioning dendritic cells and expanding regulatory T cells.^59^ These comparisons suggest that distinct helminths assign different functional weights to direct parasite effectors and microbiota-dependent circuits. In the phenotypes tested here, the *L. murinus*–ILA–AhR pathway functions as a dominant functional relay and major convergence node for Treg/Th17 remodeling and inflammatory disease tolerance, while parasite-derived pathways may regulate additional dimensions of the *T. spiralis*–host interaction. Structural modeling and SPR using human AhR established direct receptor engagement by ILA within a structurally defined human receptor framework,^60^ complementing pharmacological and genetic AhR loss-of-function in mice that established the receptor's functional requirement *in vivo*. Thus, the strong AhR dependence in our models identifies the pathway on which the defined metabolic and protective outputs examined here critically converge.

Several questions now define the next phase of this work: which of the candidate mucosal, antimicrobial, and metabolic niche-selecting mechanisms is necessary and sufficient for *L. murinus* enrichment; which AhR-expressing cell populations are necessary and sufficient for distal protection; whether additional infection-enriched indoles cooperate with ILA; and whether analogous metabolic circuits operate across human helminth infections and non-helminth inflammatory diseases. Resolving these layers will determine the generality and therapeutic window of the pathway. More broadly, our study reframes helminth immunoregulation as an ecological relay in which a metazoan parasite edits bacterial metabolism to control host immune state. This mechanism provides a conceptual basis for systemic effects that extend beyond the infected tissue and offers a route to separate beneficial disease tolerance from parasite exposure. The *T. spiralis*–*L. murinus*–ILA–AhR circuit therefore represents both a mechanistic principle of host–microbe–parasite coadaptation and a tractable molecular entry point for limiting inflammatory tissue damage.

### Limitations of the study

This study has several limitations. Although antibiotic perturbation, fecal transplantation, and supplementation experiments support a causal contribution of the gut microbiota, the present study does not quantitatively partition the entire *in vivo* ILA pool among individual taxa. The convergence of species-level *L. murinus* enrichment, culture-associated ILA accumulation, and *in vivo* supplementation nevertheless supports *L. murinus* as a functional contributor to the infection-associated ILA pool; additional ILA-producing taxa or community metabolic interactions may contribute in parallel. The relative contributions of mucosal remodeling, antimicrobial ecological filtering, and metabolic substrate restructuring to the selective enrichment of *L. murinus* remain to be quantitatively partitioned. This limitation highlights a function-centered framework, in which conserved ILA-producing capacity may be more relevant for translation than the presence of a specific bacterial species. In addition, pharmacological inhibition and whole-body AhR deletion do not resolve the relative contributions of epithelial, myeloid, and lymphoid AhR signaling, or establish whether Treg/Th17 remodeling is required for distal tissue protection. Finally, translational inference is limited by the use of a murine-adapted bacterium, preventive ILA administration in K18-hACE2 mice, and observational reanalysis of a human COVID-19 cohort. Further studies using defined microbial communities, cell-specific AhR models, therapeutic dosing paradigms, and prospective human cohorts will be required.

## Supplementary Material

R1_Supplemental_material_1_13_Oct_2026_09_21_AU.docx Supplementary Material

## Data Availability

Metagenomic sequencing data are available through the NCBI BioProject database under accession number PRJNA1321505. This study also re-analyzed publicly available COVID-19 metabolomic, proteomic and clinical data from Su et al.^36^ No custom code was generated. Additional information required to reanalyze the data reported here is available from the lead contact on reasonable request.
