# Supplementary material for: A helminth–microbiota metabolic relay promotes disease tolerance
Source: Gut Microbes. 2026 Oct 15;18(1):2748132. doi: 10.1080/19490976.2026.2748132 (PMC13682397; doi:10.1080/19490976.2026.2748132)
Supplement: Supplementary file 1 [file KGMI_A_2748132_SM7387.docx]

**Supplementary material**

**A helminth–microbiota metabolic relay promotes disease tolerance**

Ruohang Sun^1, 7^, Yi Liu^1, 2, 7^, Yang Wang^1^, Nuo Xu^1^, Xiaoxiao Ma^1^, Qingbo Lv^1^, Xiufeng Zhao^3^, Pascal Boireau^4,5^, Zeming Wu^6^, Mingyuan Liu^1^, Xiaolei Liu^1, *^, Xuemin Jin^1, *^

* Correspondence: Xiaolei Liu^*^, Xuemin Jin ^*^

Email: jinxm666@163.com (XJ) and liuxlei@163.com (XL).

**Supplementary Figures**

**Supplementary Fig. S1.**

**Figure S1. Ts** **infection remodels host tryptophan metabolism.**

(A) Principal Coordinates Analysis (PCoA) showing distinct serum metabolic profiles between control and Ts-infected mice.

(B) Heatmap of differentially abundant serum metabolites.

(C) The levels of L-Tryptophan in the feces from control and Ts-infected mice.

(D) The levels of L-Tryptophan in the serum from control and Ts-infected mice.

Data are presented as mean ± SD. Statistical analysis was performed using an unpaired Student's t-test (c and d). ** p < 0.01.

**Supplementary Fig. S2.**

**Figure S2. Ts infection promotes the indole derivative pathway.**

(A-H) The levels of tryptophan metabolites in the feces from control and Ts-infected mice.

(I-M) The levels of tryptophan metabolites in the serum from control and Ts-infected mice.

Data are presented as mean ± SD. Statistical analysis was performed using an unpaired Student's t-test. ns, no statistical significance, *, p < 0.05, **, p < 0.01.

**Supplementary Fig. S3.**

**Figure S3. Ts drives AhR-dependent modulation of the Treg/Th17 balance.**

(A-E) qPCR analysis of AhR Cyp1a1 and Treg/Th17-associated genes expression in the intestinal tissue of mice (n=6).

Data are presented as mean ± SD. Statistical significance was determined by one-way ANOVA followed by Tukey multiple comparison test. **, p < 0.01, *** p < 0.001, **** p < 0.0001.

**Supplementary Fig. S4.**

**Fig. 1G, 1I, 1K, 1M**

**Figure S4. Flow cytometric gating strategies were used in this study.** Gating strategies for all flow cytometric analyses presented in the main figures of this manuscript.

**Supplementary Fig. S5.**

**Figure S5. Ts modulates tryptophan metabolism via the microbiota**

(A) Quantification of fecal bacterial load by 16S rRNA gene qPCR. (n=6)

(B) α diversity (Obs index, Shannon index, Simpson index and Chao 1) in the microbiota from the ABX and ABX+Ts-infected mice.

(C) Principal coordinates analysis (PCoA) plot of metagenomic sequencing from ABX and ABX+Ts-infected mice.

(D) Phylum and Family-level median relative abundances.

(E) Principal coordinates analysis (PCoA) plot of metabolomic target analyses from ABX and ABX+Ts-infected mice.

(F) Heatmap of differentially abundant fecal metabolites.

(G-K) qPCR analysis of AhR and Cyp1a1 and Treg/Th17-associated genes expression in the intestinal tissue of mice from different treatment groups. (n=6).

(L) Mice were orally administered antibiotics daily for two weeks, followed by oral infection with 500 TsML. At 7 dpi, intestinal adult worms were recovered from mice in each group to determine the *T. spiralis* worm burden (n = 6).

(M-Q) qPCR analysis of AhR and Cyp1a1 and Treg/Th17-associated genes expression in the intestinal tissue of mice from different treatment groups. (n=6).

Data are presented as mean ± SD and were analyzed by Student's t-test (G-L) or one-way ANOVA with Tukey's test (A and M-Q). ns, no statistical significance, ****, p <0.0001.

**Supplementary Fig. S6.**

**Figure S6. Ts infection reshapes the host gut microbiota.**

(A) α diversity (Obs index, Shannon index, Simpson index and Chao 1) in the microbiota from the controls and Ts-infected mice.

(B) Venn of microbiota.

(C) Phylum and Family-level median relative abundances.

**Supplementary Fig. S7.**

**Figure S7. *L. murinus* and ILA supplementation activates downstream tryptophan metabolism.**

(A-F) qPCR analysis of AhR, Cyp1a1 and Treg/Th17-associated genes expression in the intestinal tissue of mice from control, *L.murinus* (A-C) and ILA (D-F) groups (n=6).

(G-J) In vitro effects in colonic organoids. Dose-dependent upregulation of AhR and Cyp1a1 mRNA in organoids treated with *L. murinus* (1×10^6^ CFU/mL) or ILA (2 mM) for 48 h, which was markedly attenuated by co-treatment with the AhR antagonist CH223191(10 μM). (n=6)

(K and L) qPCR analysis of AhR and Cyp1a1 mRNA expression in the organoids treated with different doses of ILA (n=6).

Data are presented as mean ± SD and were analyzed by Student's t-test (A-F) or one-way ANOVA with Tukey's test (G - L). *, p < 0.05, **, p < 0.01. *** p < 0.001, **** p < 0.0001.

**Supplementary Fig. S8.**

**Figure S8. *L. murinus* and ILA reduce the inflammation of organoids.**

(A and B) ELISA analysis of TNF-α, IFN-γ in the organoids culture supernatant (n=6).

(C and D) qPCR analysis of TNF-α, IFN-γ mRNA expression in the organoids (n=6).

Data are shown as individual data points and mean ± SD. Statistical significance is calculated using Tukey multiple comparison tests. **** p < 0.0001.

**Supplementary Tables**

**Table S1. qRT-PCR primers were used in this study.**

| **Target genes** | **Forward primer sequence (5´→ 3´)** | **Reverse primer sequence (5´→ 3´)** |
| --- | --- | --- |
| AhR | GAGCACAAATCAGAGACTGG | TGGAGGAAGCATAGAAGACC |
| Cyp1a1 | GTTAACCATGACCGGGAACT | GTGACCTTCTCACTCAAGCG |
| TGF-β | TGATACGCCTGAGTGGCTGTCT | CACAAGAGCAGTGAGCGCTGAA |
| Foxp3 | GGCAGAGGACACTCAATGAAAT | TCTCCACTCGCACAAAGCAC |
| IL-17A | TACCTCAACCGTTCCACGTC | TTTCCCAACCGCATTGACACA |
| β-actin | GGCTGTATTCCCCTCCATCG | CCAGTTGGTAACAATGCCATGT |
